# Treatment options of traditional Chinese patent medicines for dyslipidemia in patients with prediabetes: A systematic review and network meta-analysis

**DOI:** 10.3389/fphar.2022.942563

**Published:** 2022-08-29

**Authors:** Li Jiang, Shidong Wang, Jinxi Zhao, Chieh Chien, Yaofu Zhang, Guanxun Su, Xiaoyu Chen, Dechao Song, Yu Chen, Weijun Huang, Yonghua Xiao, Yandong Cao, Zixian Hu

**Affiliations:** ^1^ Section II of Endocrinology and Nephropathy Department of Dongzhimen Hospital Affiliated to Beijing University of Chinese Medicine, Beijing, China; ^2^ Department of Health Sciences and Medicine, University of Lucerne, Lucerne, Switzerland; ^3^ Key Laboratory of Chinese Internal Medicine of Ministry of Education and Beijing, Dongzhimen Hospital Affiliated to Beijing University of Chinese Medicine, Beijing, China

**Keywords:** traditional Chinese patent medicine, guidelines, prediabetes, dyslipidemia, network meta-analysis

## Abstract

**Objective:** To compare the clinical efficacy and safety of SIX Traditional Chinese Patent Medicines (TCPM) recommended by guidelines in improving lipids for patients with prediabetes by network meta-analysis.

**Methods:** Randomized controlled trials of 6 TCPM in the treatment of prediabetes were searched systematically in various databases. After extracting effective data, the risk of bias was assessed using Review Manager 5.3 and Cochrane Collaboration Systems Evaluator’s Manual. Network meta-analysis was performed using STATA 15.0 based on the frequency statistical model. The effect size and credibility of the evidence for the intervention were summarized based on a minimal contextualized framework.

**Results:** A total of 27 studies involving 2,227 patients were included. Compared with lifestyle modification (LM), Shenqi + LM [SMD −0.49 (95% CI: −0.85, −0.12)] and Jinqi + LM [SMD −0.44 (95% CI: −0.81, −0.06)] showed statistically significant effect in lowering TG, Shenqi + LM [SMD −0.51 (95%CI: −0.86, −0.17)] and Jinqi + LM [SMD −0.44 (95%CI: −0.80, −0.08)] in lowering TC, Jinlida + LM [SMD −0.31 (95%CI: −0.59, −0.04)] in lowering LDL-C, Shenqi + LM [SMD 0.29 (95%CI: 0.06, 0.51)] and Jinqi + LM [SMD 0.16 (95%CI: 0.01, 0.31)] in increasing HDL-C.

**Conclusion:** For patients with prediabetes, Traditional Chinese patent medicine Jinqi and Shenqi combined with lifestyle modification were associated with a significant reduction in TG and TC, while Shenqi + LM was among the most effective. Jinlida + LM was among the least effective.

**Systematic Review Registration:** https://clinicaltrials.gov/, identifier PROSPERO(CRD42021279332).

## 1 Introduction

Prediabetes refers to an intermediate stage of dysglycemia along the continuum from normo-glycemia to diabetes. It is characterized by mild impaired fasting blood glucose (IFG) and/or impaired glucose tolerance (IGT) and clinically assessed by fasting blood glucose (FBG), glycosylated hemoglobin level (HbA1C), and 2-h post-load blood glucose (2hBG) (Echouffo-Tcheugui and Selvin, 2021). The increasing prevalence of prediabetes globally is a major public health concern and exacerbates the growing epidemic of diabetes and its complications. According to the “Prevalence and Treatment of Diabetes in China, 2013–2018” issued by Chinese official institutions, the estimated prevalence of prediabetes was 35.7% in 2013 and 38.1% in 2018, bringing a huge potential burden to the health system (Xu et al., 2013). Dyslipidemia, the independent risk factor for coronary atherosclerosis, was shown to be a high-risk factor for cardiovascular disease associated with diabetes. Clinical studies have recently revealed that Traditional Chinese Patent Medicines (TCPM) played a positive role in improving the lipid profile and relieving symptoms of prediabetes. So, several TCPMs have been clearly recommended as the important intervention for prediabetes in Chinese domestic guidelines (Endocrinology and Metabolic Diseases Committee of the Chinese Physicians Association, Integrated Chinese and Western Medicine Branch, 2021; Chinese Diabetes Society, 2021), which are namely Shen qi jiang tang capsule/granule (Shenqi), Tian mai xiao ke tablet (Tianmai), Tian qi jiang tang capsule (Tianqi), Jin qi jiang tang tablet (Jinqi), Jin li da granule (Jinlida), and Tang mai kang granule (Tangmaikang) (Jiang et al., 2022).

In the traditional Chinese medicine (TCM) context, diabetes and pre-diabetes are both diseases caused by Fire toxin, which injures Qi and damages the normal function of the body. Qi is the essential substance to maintain fluid metabolism. Qi deficiency would cause clinical manifestations like thirst and fatigue in these patients (Pang and Ni, 2019). The included TCPM all followed the basic understanding of traditional context and contained mainly herbs that have the effect of clearing Fire and benefiting Qi, thus showing some positive significance in clinical research studies and getting recommended in experts’ advice and official guidelines (Xia et al., 2020). Until now, although there were some meta-analyses focusing on the clinical effectiveness and safety of TCPM for treating prediabetes (Pang et al., 2017; Pang et al., 2018; Jiang et al., 2022), the general shortcomings still existed: 1) the main outcome was blood glucose indicators, such as glycosylated hemoglobin, FBG, and PBG, with insufficient emphasis on lipids. 2) The number of literatures covered was limited, the sample size was insufficient, and the heterogeneity among different comparisons was large; 3) in recent add-on research studies, there was still a lack of comparative efficacy between the various types of TCPM.

Network meta-analysis (NMA) is a method which has been applied widely to assess all the available medications and rank different therapies through the transitivity of the same control within a consistent framework, even though when there was no direct comparison in head-to-head trials. Therefore, this study used the NMA to compare the effectiveness and safety of the aforementioned 6 TCPM in improving the lipid profile, in order to provide an evidence-based reference for clinical use in the supplementary therapy for prediabetes.

## 2 Methods

This article was reported following the guidelines of the Cochrane Multiple Interventions Methods Group and PRISMA Extension Statement (Hutton et al., 2015) and the checklist was shown in Supplementary File S1. The protocol for the research was submitted to the International Prospective Register of Systematic Reviews (PROSPERO) (CRD 42020180045). The web-based registration scheme could be found in Supplementary File S2. The introduction and discussion section of the article was elaborated with reference to the General requirements for developing, conducting, and reporting pharmacological research on medicinal plants and natural products (phytopharmacology) (Heinrich et al., 2020). The summary table describing the composition of the preparations and how these were reported in the original studies was structured following the principles described in the Four Pillars of Ethnopharmacology.

### 2.1 Inclusion criteria

The research studies were considered if they matched the following criteria: 1)randomized controlled trials (RCTs) with available data on blood lipids, including triglyceride (TG), total cholesterol (TC), low-density lipoprotein cholesterol (LDL-C), high-density lipoprotein cholesterol (HDL-C). LDL-C was chosen as the primary outcome for its independent predicting effect on the risk of ASCVD in individuals or populations. The others were secondary outcomes; 2) patients with prediabetes (≥18 years old) according to the various definitions indicated by authoritative academic institutions, including the ADA (American Diabetes Association, 2018), the WHO (WHO, 1999), and the Chinese Diabetes Society (CDS) (Chinese Diabetes Society, 2021); 3) treatments of interest in the experimental group contained 6 kinds of TCPM (Shenqi, Tianqi, Jinlida, Tianmai, Jinqi, and Tangmaikang). The TCPM is defined as the Chinese medical products made from herbal medicine and processed into certain dosage forms. The formula is formed according to prescribed prescriptions under the guidance of the theory of TCM. The manufacturing is put in line with specified pharmaceutical technology based on the PRC Pharmacopoeia’s monograph (Pang et al., 2017). Interventions in the control group were not limited (LM, placebo, oral hypoglycemic drugs, or their combination); 4) treatment duration of the study which includes HbA1c should be more than 12 weeks.

### 2.2 Exclusion criteria

The clinical studies were excluded with the following features: 1) Interventions in the experimental group contained oral hypoglycemic drugs. 2) Interventions in the experimental group included other TCM such as no-decoction pellets and water extracts. 3) The ingredients of applied TCPM were not completely stated or inconsistent with the description in Table 2. 4) Lipid-lowering drugs such as statins, fibrates, and monacolins were used in either group; 5) Common clinical practices in TCM such as acupuncture, cupping, Gua Sha and Tui Na were combined in either group; 6) Data of outcome indicators were still unavailable after contacting authors.

### 2.3 Study identification

A comprehensive search strategy whose search string followed the PICOS method was developed to find peer-reviewed published studies. The English database like PubMed, EMBASE, Cochrane Library, Web of Science, and Clinical trials, and the Chinese database like Sinomed, CNKI, and WanFang. and VIP was used for article retrieval up to July 2022. Two prior systematic reviews (Pang et al., 2017; Pang et al., 2018) which were searched ended in September 2017 have been updated and referred to. Additionally, we also consulted with experts to identify candidate studies. The full electronic search strategy for Pubmed and CNKI was presented in Supplementary File S3.

### 2.4 Data extraction

Two independent reviewers (Li Jiang and Chieh Chien) screened the literature and extract data separately with reference to the Cochrane Handbook (Higgins et al., 2019). Information was checked and adjudicated independently by an additional investigator (Yaofu Zhang) until agreement was achieved. A pre-tested standard data extraction form will be designed specifically for this review. It contained the following base entries: the first author of the article, year of publication, sample size, age and gender distribution of subjects, diagnostic criteria, mode of intervention (including dose, frequency, usage), duration of intervention, lipid-related outcome indicators, the plan of randomization and concealment and specific reports of adverse reactions, etc. Δ indicates the change in indicators before and after the intervention.

### 2.5 Quality assessment

The methodological quality of eligible RCTs was examined in Software Review Manager 5.3. The assessment tool standard covers 7 aspects: random sequence generation; allocation concealment; blinding of participants and personnel; blinding of outcome assessment; incomplete outcome; selective reporting; other biases. For each study, the aforementioned seven items were evaluated as “low bias”, “high bias”, and “unclear”. The differences were reviewed by the third member, and finally determined and drew the bias risk map after discussion. The Grading of Recommendations Assessment, Development, and Evaluation (GRADE) framework was used to assess the quality of evidence contributing to each estimated network, which namely have four grades, high, Moderate, low, and very low (Liu, 2022). The seven assessment domain of each contrast include the risk of bias, inconsistency, the indirectness, imprecision, publication bias, intransitivity, and incoherence in the GRADE framework for NMA (Puhan et al., 2014; Brignardello-Petersen et al., 2021).

### 2.6 Statistical analysis

We used frequentist network meta-analysis in software STATA 15.0 (Bhatnagar et al., 2014). For direct comparisons, the pooled analysis of the overall effect size for continuous variable outcome was presented as standardized mean difference (SMD) and an associated 95% confidence interval (CI) using the DerSimonian and Laird random-effects methods (DerSimonian and Laird, 1986). The assumption of consistency in the whole analytical network was conducted in a design-by-treatment approach. The loop-specific approach was used to evaluate the presence of inconsistency in network meta-analysis models locally. If no loop is formed, the node-splitting analysis can be applied (Caldwell et al., 2005). Heterogeneity was assessed by the Chi-squared test and the I^2^ index (Higgins et al., 2019), which was considered significant if I^2^ > 75% (Page et al., 2021). Sensitivity analyses, meta-regression, subgroup analyses, and a random-effects model were utilized if the heterogeneity was significant. Otherwise, the fixed-effects model was selected. Meta-regression was performed on the treatment duration (≤3, 3–6, ≥6 months), the type of TCPM, the control group (LM, LM + placebo, LM + oral hypoglycemic drugs), the diagnostic criteria (WHO, ADA, and CDS), the risk of bias (high, not high, unclear) and the baseline of LDL-C (ideal level: < 2.6, appropriate level: 2.6–3.4, pathologically elevated: 3.4 mmol/L) (Joint committee issued Chinese guideline for the management of dyslipidemia in adults, 2016). The forest plot and the league table was used to display the pairwise comparison in NMA. The surface under the cumulative ranking curve (SUCRA) would be used to estimate ranking probabilities for all treatments in order to create a treatment hierarchy (Salanti et al., 2011). The minimally contextualized framework was developed according to the result of the SUCRA and GRADE evaluation (Brignardello-Petersen et al., 2020). In addition, the correction funnel plot was drawn to assist in determining whether there is publication bias or a small sample effect among included literature.

## 3 Result

### 3.1 Baseline characteristics

**FIGURE 1:**
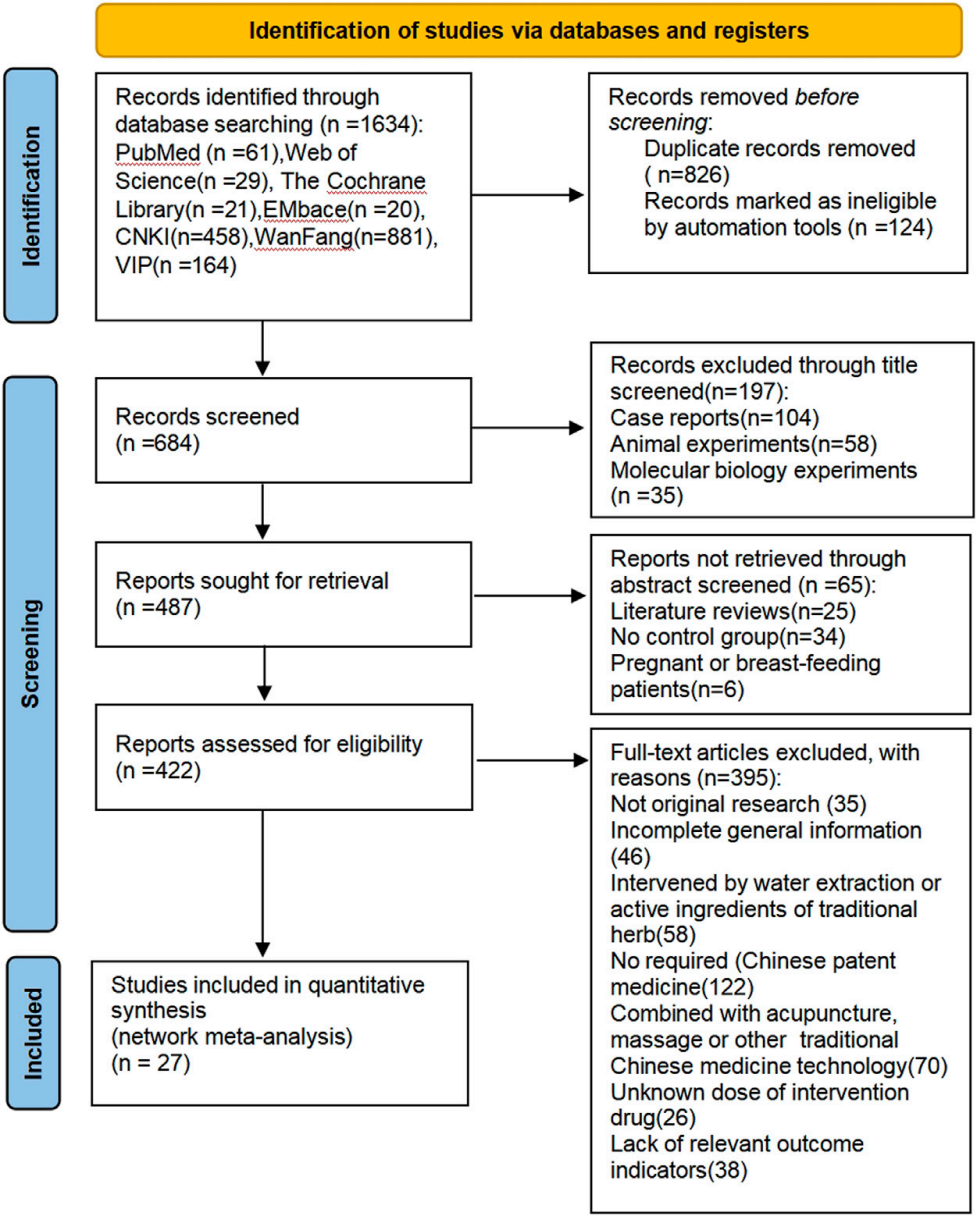
Study selection process.

A final total of 27 articles were included in the NMA. The process was shown in Figure 1. All trials were conducted in China and published between 2002 and 2018, corresponding to 2,227 adults (1,139 in the treatment group and 1,088 in the control group). Both groups were based on a lifestyle modification (LM), with the addition of TCPM in the treatment group and the addition of oral hypoglycemic drugs or placebo or blank to the control group. Among them, there were five articles related to Shenqi, two related to Tianmai, three related to Tianqi, five related to Jinqi, six related to Jinlida, and six related to Tangmaikang. LDL-C has been reported in 18 type of research, HDL-C in 17 researches, and TG, and TC in 27 researches. The basic characteristics of the included studies was shown in Table 1. The source, ingredients, quality control, and chemical analysis of the included TCPM were shown in Table 2. A total of 40 preclinical studies investigated the lipid or glucose-lowering effects of 6 TCPM in prediabetes or T2DM. The beneficial effects and potential mechanisms are summarized in Table 3 **.**

**TABLE 1:** Characteristics of the included studies.

Study ID (Author + time)	Sample size (M/F)	Age (year)	Diagnostic criteria	Intervention Treatment group	Control group	Duration (month)	Outcome measure
Chen (2005)	T:46 (22/14) C:32 (18/14)	T:53.1 ± 10.1C:52.5 ± 9.3	WHO 1999	Shenqi capsule (0.7 g tid)+LM	LM	12	①②
Lin and Hu (2007)	T:29 (unclear) C:29 (unclear)	T:53.6 ± 4.4C:52.9 ± 5.8	WHO 1999	Shenqi granule (3 g bid)+LM	LM	6	①②
Tian and Li (2012)	T:30 (17/13) C:30 (15/15)	T:50.6 ± 6.4C:51.2 ± 6.2	WHO 1999	Shenqi granule (3 g tid)+LM	LM	6	①②③④
Yan et al. (2011)	T:25 (13/12) C:25 (14/11)	T:51.4 ± 2.2C:50.6 ± 2.4	ADA 2008	Shenqi granule (3 g bid)+LM	LM	3	①②③④
Zhao (2018)	T:45 (13/32) C:45 (15/30)	T:51.5 ± 4.8C:51.3 ± 4.3	ADA 2010	Shenqi granule (3 g tid)+LM	LM	3	①②
Dong and Wang (2015)	T:42 (18/24) C:42 (20/22)	T:52.4 ± 8.6C:54.1 ± 7.9	CDS 2013	Tianmai tablet (0.24 g bid)+LM	LM	6	①②③④
Zhang et al. (2011)	T:60 (28/32) C:60 (29/31)	—	WHO 1999	Tianmai tablet (0.24 g bid)+LM	LM + placebo	24	①②③④
Wei (2009)	T:30 (10/19) C:30 (10/21)	T:52.6 ± 6.10C:51.7 ± 5.6	WHO 1999	Tianqi capsule (8 g tid)+LM	LM + placebo	6	①②③④
Chen (2011)	T:63 (27/36) C:59 (32/27)	T:52.8 ± 10.5C:52.9 ± 10.9	WHO 1999	Tianqi capsule (8 g tid)+LM	LM + placebo	12	①②③④
Wang et al. (2011a)	T:90 (40/54) C:74 (34/40)	T:51.4 ± 8.7C:51.7 ± 9.3	WHO 1999	Tianqi capsule (8 g tid)+LM	LM + placebo	12	①②③④
Chen and Wu (2007)	T:32 (18/14) C:27 (15/12)	T:44 ± 8.6C:45 ± 9.1	ADA 1997	Jinqi tablet (2.52 g tid)+LM	LM	1	①②③④
Mao (2003)	T:32 (15/17) C:30 (14/16)	T:64.8 ± 5.4C:63.9 ± 5.8	WHO 1985	Jinqi tablet (2.94 g tid)+LM	LM	3	①②
Tan (2010)	T:42 (20/22) C:42 (21/21)	T:58.4 ± 2.1C:54.8 ± 1.6	ADA 2008	Jinqi tablet (3.36 g tid)+LM	LM	3	①②③④
Zhou and Chen (2003)	T:46 (18/28) C:42 (17/25)	T:55.6 ± 12.1C:54.0 ± 11.3	WHO 1985	Jinqi tablet (2.94 g tid)+LM	LM	12	①②④
Zhou (2002)	T:24 (9/15) C:22 (8/14)	T:45.4 ± 7.0C:46.0 ± 6.8	WHO 1985	Jinqi tablet (4.2 g tid)+LM	LM	1	①②④
Wang et al. (2011b)	T:65 (35/30) C:65 (33/32)	T:72.6 ± 4.1C:73.1 ± 3.8	WHO 1999	Jinlida granule (9 g tid)+LM	LM	6	①②③④
Cai et al. (2017)	T:60 (32/28) C:60 (30/30)	T:46.4 ± 10.6C:48.2 ± 9.6	CDS 2013	Jinlida granule (9 g tid)+LM	LM	3	①②③④
Liu (2015)	T:52 (24/28) C:49 (22/27)	T:49.6 ± 11.3C:47.9 ± 11.8	WHO 1999	Jinlida granule (9 g tid)+LM	LM	3	①②③④
Wang and Jiang (2018)	T:42 (23/19) C:37 (20/17)	T:57.1 ± 10.6C:55.6 ± 11.4	CDS 2010	Jinlida granule (9 g tid)+LM	LM	4	①②③
Yin (2016)	T:42 (22/20) C:41 (23/18)	T:47.8 ± 7.1C:48.4 ± 6.8	CDS 2010	Jinlida granule (9 g tid)+LM	LM + metformin	2	①②③④
Shi et al. (2016)	T:32 (17/15) C:29 (14/15)	T:47.1 ± 7.1C:49.9 ± 7.2	WHO 1999	Jinlida granule (9 g tid)+LM	LM	3	①②③④
Gu (2007)	T:36 (unclear) C:36 (unclear)	42.3	WHO 1999	Tangmaikang granule (5 g tid)+LM	LM	24	①②
Shen et al. (2006)	T:27 (unclear) C:28 (unclear)	55.4 ± 8.7	ADA 2003	Tangmaikang granule (5 g bid)+LM	LM	3	①②
Tao et al. (2012)	T:27 (14/13) C:28 (9/19)	T:55.1 ± 10.4C:55.4 ± 7.7	ADA 2003	Tangmaikang granule (5 g bid)+LM	LM	3	①②
Cao and Jin (2010)	T:36 (22/14) C:36 (20/16)	T:55.5 ± 2.6C:55.4 ± 2.4	ADA 2008	Tangmaikang granule (5 g tid)+LM	LM + metformin	3	①②③④
Hong et al. (2014)	T:45 (26/19) C:45 (28/17)	T:67.5 ± 5.8C:65.4 ± 6.2	WHO 1999	Tangmaikang granule (5 g tid)+LM	LM + metformin	3	①②③
Xiao et al. (2013)	T:45 (27/18) C:45 (26/19)	—	ADA 2006	Tangmaikang granule (5 g tid)+LM	LM + metformin	3	①②

Note: T: Treatment group; C: Control group; LM: lifestyle modification; ① TC (Total cholesterol); ② TG (triglyceride); ③ LDL- C; ④ HDL- C.

**TABLE 2:** Patented formulations of the Included TPCM.

Study	Formulation	Source	Species, concentration	Quality control reported? (Y/N)	Chemical analysis reported? (Y/N)
Chen (2005)	Shenqi capsule/granule	[Henan Lingrui Pharmaceutical, Co. Ltd.] SFDA approval number: Z10970002	1.Total Ginsenoside of *Panax ginseng* C.A.Mey. [Araliaceae] from stems and leaves (Renshen jing ye zaogan) 6 g	Y-Prepared according to Chinese Pharmacopoeia (2020 Edition) Chinese Pharmacopoeia Commission (2020)	Y—HPLC Yan et al. (2014), UPLC-MS/MS Zhang et al. (2021), RP-HPLC Feng et al. (2021), UPLC-Q-TOF MS Zhang et al. (2017), GC-MS Wang et al. (2021)
Lin and Hu (2007)			2.*Astragalus mongholicus* Bunge [Fabaceae] (Huangqi), 124 g		
Tian and Li (2012)			3.*Dioscorea oppositifolia* L. [Dioscoreaceae](Shanyao), 62 g		
Yan et al. (2011)			4.*Poria cocos* (Schw.) Wolf. (Fuling), 62 g		
Zhao (2018)			5.*Rehmannia glutinosa* (Gaertn.) DC. [Orobanchaceae](Dihuang), 186 g		
			6.*Ophiopogon japonicus* (Thunb.) Ker Gawl. [Asparagaceae](Maidong), 62 g		
			7.*Schisandra chinensis* (Turcz.) Baill. [Schisandraceae](Wuweizi), 62 g		
			8.*Trichosanthes kirilowii* Maxim. [Cucurbitaceae](Tianhuafen), 62 g		
			9.*Rubus chingii* Hu [Rosaceae](Fupenzi), 31 g		
			10.*Alisma plantago-aquatica subsp. Orientale* (Sam.) Sam. [Alismataceae](Zexie), 62 g		
			11.*Lycium chinense* Mill. [Solanaceae](Gouqi), 124 g		
			Made into 1,000 capsules		
Dong and Wang (2015)	Tianmai tablet	[Hebei Fuge Pharmaceutical, Co. Ltd.] SFDA approval number: Z20049007	1.Chromium picolinate, 1.6 mg	N	N
Zhang et al. (2011)			2.*Trichosanthes kirilowii* Maxim. [Cucurbitaceae](Tianhuafen), unknown dosage		
			3.*Schisandra chinensis* (Turcz.) Baill. [Schisandraceae](Wuweizi), unknown dosage		
			4.*Ophiopogon japonicus* (Thunb.) Ker Gawl. [Asparagaceae](Maidong)		
			Unknown dosage		
Wei (2009)	Tianqi capsule	[Heilongjiang Weimingtianren Pharmaceutical, Co. Ltd.] SFDA approval number: Z20063799	1.*Panax ginseng* C.A.Mey. [Araliaceae]	Y-Prepared according to the State Drug Administration standard WS-666 (Z-186) 2002, TLCS was used to determine the content of berberine hydrochloride in Huanglian and TLC was used to identify the thin layer chromatography of Nvzhenzi, Renshen, Huangqi, Huanglian and Wubeizi (Cao et al. (2009)	Y—UPLC-LTQ Orbitrap HRMS He et al. (2018)
Chen (2011)			2.*Astragalus mongholicus* Bunge [Fabaceae] (Huangqi)		
Wang et al. (2011a)			3.*Trichosanthes kirilowii* Maxim. [Cucurbitaceae](Tianhuafen)		
			4.*Ligustrum lucidum* W.T.Aiton. [Oleaceae](Nvzhenzi)		
			5*.Eclipta prostrata* (L.) L. [Asteraceae](Hanliancao)		
			6.*Coptis chinensis* Franch. [Ranunculaceae](Huanglian)		
			7.*Dendrobium nobile* Lindl. [Orchidaceae] (Shihu)		
			8.*Lycium chinense* Mill. [Solanaceae](Digupi)		
			9.*Rhus chinensis* Mill. [Anacardiaceae](Wubeizi)		
			10.*Cornus officinalis* Siebold & Zucc. [Cornaceae](Shanzhuyu)		
			Unknown dosage		
Chen and Wu (2007)	Jinqi tablet	[Tianjin Zhongxing Pharmaceutical, Co. Ltd.] SFDA approval number: Z10920027	1.*Astragalus mongholicus* Bunge [Fabaceae] (Huangqi), 513 g	Y-Prepared according to Chinese Pharmacopoeia (2020 Edition)	Y –LC-MS/MS Wang et al. (2016a), UPLC-ESI-MS Jin et al. (2018)
Mao (2003)			2.*Coptis chinensis* Franch. [Ranunculaceae](Huanglian), 343 g		
Tan (2010)			3.*Lonicera japonica* Thunb. [Caprifoliaceae](Jinyinhua), 2058g		
Zhou and Chen (2003)			Pressed into 1,000 tablets		
Zhou (2002)					
Wang et al. (2011b)	Jinlida granule	[Shijiazhuang Yiling Pharmaceutical, Co. Ltd.] SFDA approval number: Z20050845	1.*Panax ginseng* C.A.Mey. [Araliaceae](Renshen), 184.5 g	Y-Prepared according to Chinese Pharmacopoeia (2020 Edition)	Y –HPLC An et al. (2014), UV Spectrophotometry FU et al. (2022)
Cai et al. (2017)			2.*Polygonatum sibiricum* Redouté [Asparagaceae](Huangjing), 244.5 g		
Liu (2015)			3.*Atractylodes lancea* (Thunb.) DC. [Asteraceae](Chao Cangzhu), 122.2 g		
Wang and Jiang (2018)			4.*Sophora flavescens* Aiton [Fabaceae](Kushen) 100 g		
Yin (2016)			5.*Ophiopogon japonicus* (Thunb.) Ker Gawl. [Asparagaceae](Maidong), 244.5 g		
Shi et al. (2016)			6.*Rehmannia glutinosa* (Gaertn.) DC [Orobanchaceae](Dihuang), 184.5 g		
			7.Reynoutria multiflora (Thunb.) Moldenke [Polygonaceae](Heshouwu) 149 g		
			8. *Cornus officinalis* Siebold & Zucc. [Cornaceae](Shanzhuyu), 244.5 g		
			9.*Poria cocos* (Schw.) Wolf. (Fuling), 149 g		
			10.Eupatorium fortunei Turcz. [Asteraceae](Peilan), 100 g		
			11.*Coptis chinensis* Franch. [Ranunculaceae](Huanglian), 100 g		
			12.*Anemarrhena asphodeloides* Bunge [Asparagaceae](Zhimu), 122.2 g		
			13.*Epimedium sagittatum* (Siebold & Zucc.) Maxim. [Berberidaceae]		
			(Yinyanghuo), 100 g		
			*14. Salvia miltiorrhiza* Bunge [Lamiaceae](Danshen), 160 g		
			15.*Pueraria montana var.lobata* (Willd.) Maesen & S.M.Almeida ex Sanjappa & Predeep [Fabaceae](Gegen), 244.5 g		
			16.*Litchi chinensis* Sonn. [Sapindaceae](Lizhihe), 244.5 g		
			17.*Lycium chinense* Mill. [Solanaceae](Digupi), 149 g		
			Ethanol extracted and concentrated to 1000g, 9 g per granule		
Gu (2007)	Tangmaikang capsule	[Sichuan Shenghe Pharmaceutical, Co. Ltd.] SFDA approval number: Z20090557	1.*Astragalus mongholicus* Bunge [Fabaceae] (Huangqi), 200 g	Y-Prepared according to Chinese Pharmacopoeia (2020 Edition)	Y—HPLC-ELSD Zhao and Zhang (2011), HPLC-DAD Zhang and Zhao (2011)
Shen et al. (2006)			2.*Rehmannia glutinosa* (Gaertn.) DC. [Orobanchaceae](Dihuang), 216.7 g		
Tao et al. (2012)			3.*Paeonia lactiflora* Pall. [Paeoniaceae](Chishao), 216.7 g		
Cao and Jin (2010)			4.*Salvia miltiorrhiza* Bunge [Lamiaceae] (Danshen), 200 g		
Hong et al. (2014)			5.*Achyranthes bidentata* Blume [Amaranthaceae](Niuxi), 125 g		
Xiao et al. (2013)			6.*Ophiopogon japonicus* (Thunb.) Ker Gawl. [Asparagaceae](Maidong), 125 g		
			7.*Polygonatum sibiricum* Redouté [Asparagaceae](Huangjing), 125 g		
			8.*Pueraria montana var.lobata* (Willd.) Maesen & S.M.Almeida ex Sanjappa & Predeep [Fabaceae](Gegen), 125 g		
			9.*Morus alba* L. [Moraceae](Sangye), 125 g		
			10.*Coptis chinensis* Franch. [Ranunculaceae](Huanglian), 41.7 g		
			*11. Epimedium sagittatum* (Siebold & Zucc.) Maxim. [Berberidaceae]		
			(Yinyanghuo), 166.7 g		
			Water extracted to clear paste, add about 167 g of micronized silica gel, dry and put into capsules and make 1,000 capsules, 0.5 g per capsule		

**TABLE 3:** Potential mechanisms of 6 TCPM on prediabetes and T2DM *in vivo* experiments.

Formulation	References	Beneficial effects	Potential mechanisms
Shenqi capsule/granule	Shi et al. (2022)	Improving insulin sensitivity	Decreasing the mRNA expression level and serum concentration of inflammatory cytokines such as TNF-αIL-6, and IL-1β, suppressing the p-NFκB protein over-expression, up-regulating protein expression of p-Akt and GLUT2 in a rat model of insulin resistance Shi et al. (2022)
	Zhang et al. (2019a)	Kidney protection	Reducing caspase-3-positive cells in diabetic kidneys, upregulating Bcl-2 and regucalcin expressions, and reducing casp3 and Apaf1 expressions in diabetic rats Zhang et al. (2019a)
Tianmai tablet	Wang et al. (2016b)	Improving insulin sensitivity	Decreasing IRS-1, IRS-2, PI3-K p85α, and AKT2 gene expression and also IRS-1, IRS-2, PI3-K, AKT2, and p-AKT2 protein expression levels through the PI3K/AKT pathway in diabetic rats Wang et al. (2016b)
	Zhang et al. (2014a)	Reducing fasting glucose level	Decreasing levels of forkhead box O3 (FoxO3), phosphoenolpyruvate carboxykinase 2 (Pck2), and protein tyrosine phosphatase 1B (Ptp1b), increasing v-akt murine thymoma viral oncogene homolog 1 (Akt1) and insulin receptor substrate 2 (Irs2) through insulin signaling pathway in diabetic rats Zhang et al. (2014a)
	Zhang et al. (2014b)	Activating insulin synthesis	Increasing the expression of miR-375 and miR-30d in diabetic rats Zhang et al. (2014b)
Tianqi capsule	Zhang et al. (2010)	Improving glucose metabolism	Down-regulating the apolipoprotein E, apolipoprotein A-I, Ig gamma-2A chain C region, up-regulating transthyretin (TTR), haptoglobin (Hp), serum amyloid p-component (SAP) and prothrombin in diabetic rats Zhang et al. (2010)
	Li et al. (2013)	Preventing diabetes	Reducing the “G" allele frequencies of rs1142345 (A>G) in the thiopurine S-methyltransferase (TPMT) gene in prediabetic patients Li et al. (2013)
	Qian et al. (2012)	Improving insulin sensitivity	Up-regulating the expression of IRS-1 in the liver and IRS-2 in the skeletal muscles of the KK-Ay mice Qian et al. (2012); inhibiting the phosphorylation of JNK, ERK1/2, and p38 Liu et al. (2017); lowering the circulating T helper 17 (Th17) frequencies, serum interleukin-17 (IL-17) and interleukin-23 (IL-23) levels in diabetic SD rats Lv et al. (2017)
	Liu et al. (2017)		
	Lv et al. (2017)		
	Liu et al. (2017)	Increasing glucose uptake and glycogen synthesis	Elevating the insulin-stimulated glucose uptake with upregulated phosphorylation of AKT in PA-induced insulin resistant L6 myotubes Liu et al. (2017); downregulating miRNA-29b and targeting AKT Zhao et al. (2012); increasing the expression of AMPK in the liver and muscular tissues and the GLUT-4 in the skeletal muscles, reversing the decreased glycogen level in liver Qian et al. (2012); Gao et al. (2014)
	Zhao et al. (2012)		
	Qian et al. (2012)		
	Gao et al. (2014)		
	Qian et al. (2012)	Enhancing lipid metabolism	Increasing the expression and tyrosine phosphorylation of AMPK Qian et al. (2012); decreasing the expression of acetyl CoA carboxylase (ACC), fatty acid synthase (FAS), and hormone-sensitive lipase (HSL) in diabetic KK-Ay mice Zhao et al. (2012); Liu et al. (2017)
	Zhao et al. (2012)		
	Liu et al. (2017)		
Jinlida granule	Zhou et al. (2022)	Enhancing lipid metabolism	Increasing the expression of the thermogenic protein, UCP1, in the beige adipose tissue of mice, inhibiting the expression of miR-27a in X9 cells thereby promoting thermogenesis in beige adipocytes Zhou et al. (2022); activating the brown adipose tissue thermogenesis via enhancement of mitochondrial biogenesis and fatty acid oxidation metabolism Zhang et al. (2019b)
	Zhang et al. (2019b)		
	Wang et al. (2018)	Improving dysfunction of Hypothalamic-Pituitary-Thyroid Axis	Increasing the levels of serum T3 and T4, TR mRNA in liver tissue, TSHR, and NIS mRNA in thyroid tissue, decreases the levels of Dio1 mRNA, pI-κB, pNF-κB, TNFα and IL-6 in diabetic rats Wang et al. (2018)
	Jin et al. (2015)	Improving insulin sensitivity	Increasing the expression of insulin receptor substrate (IRS-1) mRNA and protein, alleviating the expression of diacylglycerol acyltransferase (DGAT) in skeletal muscle Jin et al. (2015); increasing AMPK and acetyl-CoA carboxylase (ACC) phosphorylation in skeletal muscle; reducing hepatic oxidative stress through reducing phosphorylation protein levels of JNK and p38MAPK Liu et al. (2015); Zang et al. (2015)
	Zang et al. (2015)		
	Liu et al. (2015)		
Tangmaikang granule	Chen et al. (2017)	Reducing insulin resistance	Reducing hepatic lipid accumulation and lowering levels of serum inflammatory factor CRP Chen et al. (2017)

### 3.2 Bias assessment

**FIGURE 2:**
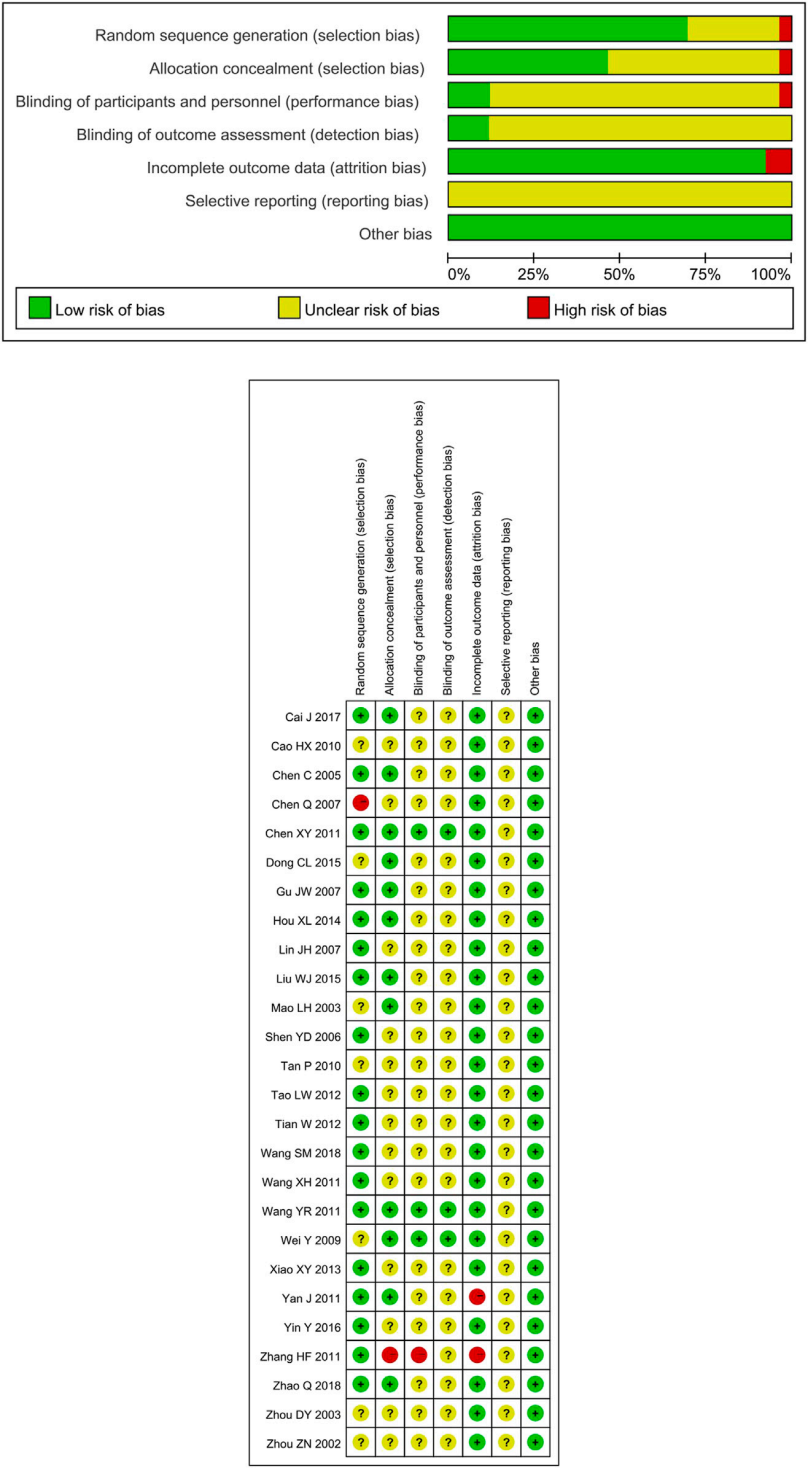
Risk of bias.

The 27 included studies all used a randomized method, 17 using random number tables generated from random sequences, and two using systematic randomization (Mao, 2003; Wang et al., 2011a), and were therefore rated as “low risk”; one study was randomized according to the order of attendance, which was pseudo-randomized and rated as “high risk”. The remaining seven studies did not mention a specific randomized method and were therefore rated as “unclear risk”. Three studies were double-blinded with a placebo (Wei, 2009; Wang et al., 2011a; Chen, 2011) and were all rated as “low risk.” The remaining 23 studies did not report allocation concealment or blinded set-up and were therefore rated as “unclear risk.” Two studies (Yan et al., 2011; Zhang et al., 2011) reported missing visits and had incomplete outcome data, and were therefore rated as “high risk.” The remaining 25 studies had no missing data and were rated as “low risk.” None of the study protocols were first registered or could not be obtained, therefore the existence of selective reporting could not be determined and was reported as “unclear risk.” There exited no other bias in included literature. The bias assessment was shown in Figure 2.

### 3.3 GRADE assessment

The GRADE judgment was incorporated and the analyses showed the quality was low or very low for most of the comparisons. As for the primary outcome, LDL-C, no direct comparison showed significant results statistically in the total of eight comparisons, and all of them were evaluated as very low except Shenqi + LM vs. LM and Jinqi + LM vs. LM which were evaluated as low. For TG and TC, two direct comparisons showed significant results statistically (Shenqi + LM vs. LM and Jinqi + LM vs. LM) in a total of nine comparisons, and they were evaluated as moderate and low quality. For HDL-C, two direct comparisons showed significant results statistically (Tianmai + LM vs. placebo + LM and Jinqi + LM vs. LM) in the total of eight comparisons, and they were evaluated as very low and low. The detailed GRADE assessment was presented in Supplementary File S4.

### 3.4 Pairwise meta-analysis

**FIGURE 3:**
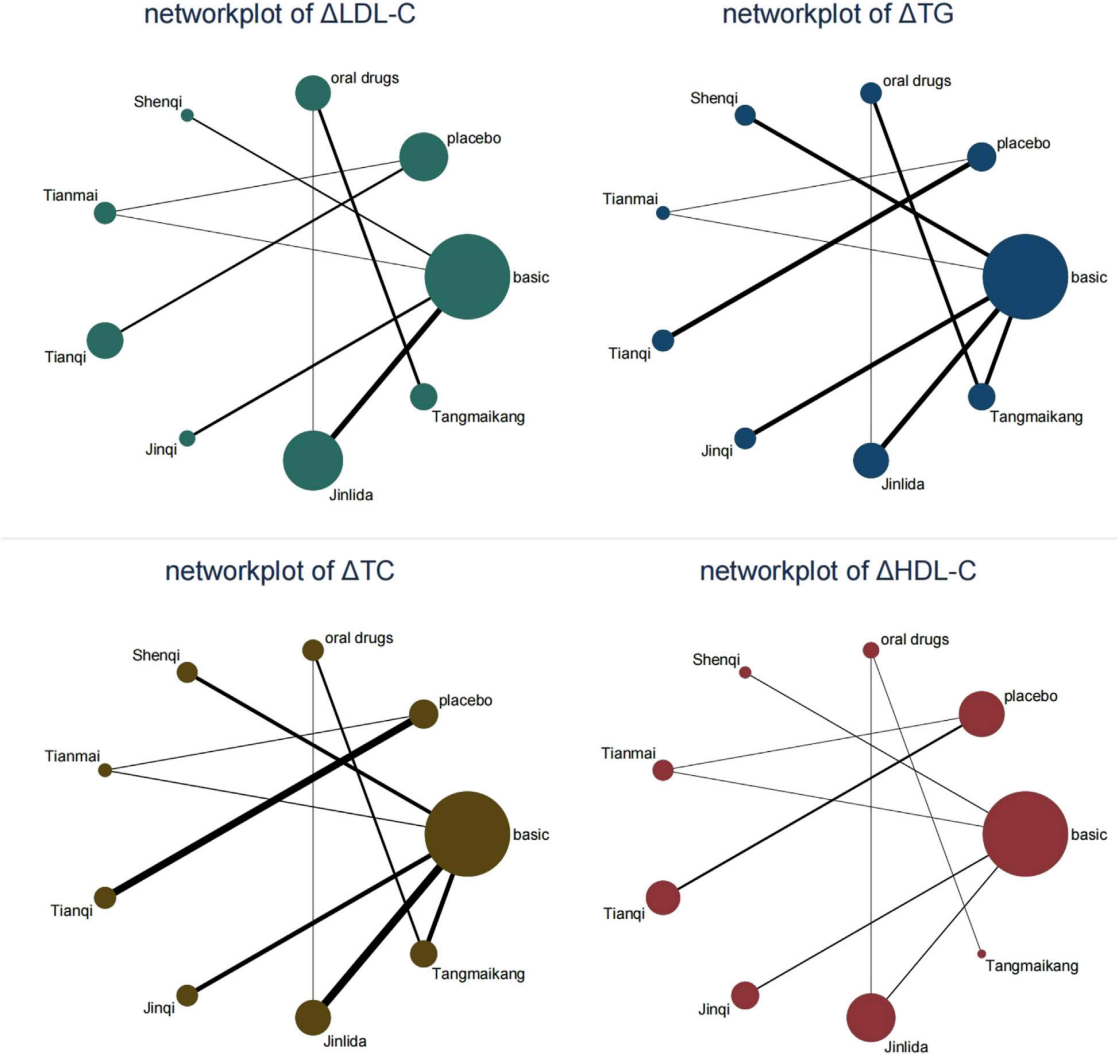
Network evidence plots. Legend: “basic” means the basic lifestyle modification without any drugs. “oral drug” means oral hypoglycemic drugs (metformin in the included studies) based on the lifestyle modification.

Network evidence plots were made based on direct comparative relationships, as shown in Figure 3, with the node representing a certain intervention. The size of the node represented the total number of people in each study, and the thickness of the line represented the standard error of SMD or logarithmic OR. The relationship between the interventions did not form a closed loop and therefore no loop inconsistency testing was required. 27 studies (Zhou, 2002; Mao, 2003; Zhou and Chen, 2003; Chen, 2005; Shen et al., 2006; Chen and Wu, 2007; Gu, 2007; Lin and Hu, 2007; Wei, 2009; Cao and Jin, 2010; Tan, 2010; Wang et al., 2011a; Wang et al., 2011b; Chen, 2011; Salanti et al., 2011; Yan et al., 2011; Zhang et al., 2011; Tao et al., 2012; Tian and Li, 2012; Dong and Wang, 2015; Liu, 2015; Shi et al., 2016; Yin, 2016; Cai et al., 2017; Wang and Jiang, 2018; Zhao, 2018; Brignardello-Petersen et al., 2020) reported TG and TC with a total arm size of 54 and 2,227 patients. 18 studies (25, 26, 28–33, 35, 39–43, 47–49] reported LDL-C with a total arm size of 36 and 1,633 patients. 17 studies (Zhou, 2002; Mao, 2003; Zhou and Chen, 2003; Chen, 2005; Shen et al., 2006; Chen and Wu, 2007; Lin and Hu, 2007; Wei, 2009; Tan, 2010; Wang et al., 2011b; Chen, 2011; Yan et al., 2011; Zhang et al., 2011; Dong and Wang, 2015; Liu, 2015; Wang and Jiang, 2018; Zhao, 2018) reported HDL-C with a total arm size of 34 and 1,508 patients.

### 3.5 Network meta-analysis

#### 3.5.1 Primary outcome

**FIGURE 4:**
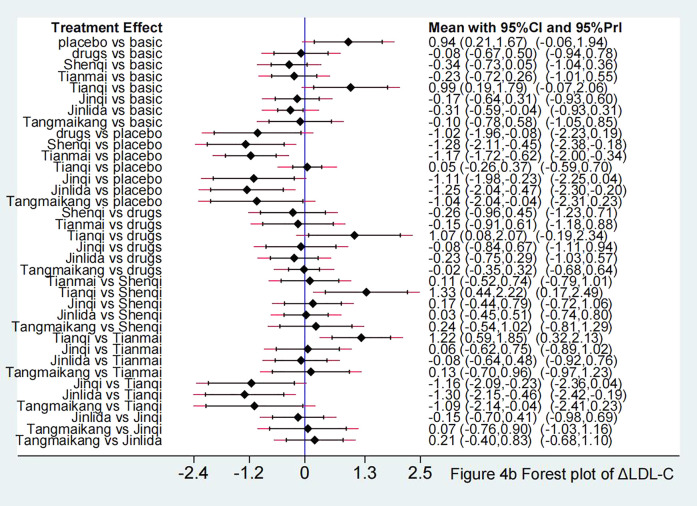
**(A)** Forest plot in ΔLDL-C.

Compared with LM, Jinlida + LM [SMD −0.31 (95%CI: −0.59, −0.04)] showed a statistically significant effect in lowering LDL-C. Compared with placebo + LM, except for Tianqi + LM [SMD 0.05 (95%CI: −0.26, 0.37)], other 5 TCPM showed statistically significant effect in lowering LDL-C, with SMD fluctuating between −1.28 (95%CI: −2.11, −0.45) (Shenqi + LM) to −1.04 (95%CI: −2.04, −0.04) (Tangmaikang + LM). Detailed comparative results are shown in Figure 4 and Table 4A.

**TABLE 4A:** The league table of ΔLDL-C and ΔHDL-C.

Comparisons for *Δ*LDL-C (bottom left) and *Δ*HDL-C (upper right) of the 6 TPCM.								
basic	**0.61 (0.40, 0.93)**	0.99 (0.72, 1.35)	**1.33 (1.07, 1.67)**	1.05 (0.80, 1.39)	**0.63 (0.39, 0.99)**	**1.17 (1.01, 1.36)**	1.10 (0.96, 1.27)	1.30 (0.83, 2.02)
0.39 (0.19, 0.81)	placebo	1.61 (0.96, 2.72)	**2.18 (1.35, 3.51)**	**1.72 (1.25, 2.35)**	1.02 (0.84, 1.24)	**1.91 (1.22, 2.99)**	**1.80 (1.16, 2.81)**	**2.12 (1.15, 3.90)**
1.09 (0.60, 1.95)	**2.78 (1.09, 7.11)**	Oral drugs	1.35 (0.92, 1.98)	1.06 (0.70, 1.61)	0.63 (0.36, 1.11)	1.18 (0.84, 1.67)	1.12 (0.85, 1.47)	1.31 (0.95, 1.80)
1.40 (0.95, 2.07)	**3.60 (1.57, 8.26)**	1.29 (0.64, 2.61)	Shenqi	0.79 (0.55, 1.13)	**0.47 (0.28, 0.79)**	0.88 (0.67, 1.15)	0.83 (0.64, 1.08)	0.97 (0.59, 1.60)
1.26 (0.77, 2.05)	**3.22 (1.86, 5.58)**	1.16 (0.54, 2.49)	0.90 (0.48, 1.67)	Tianmai	0.59 (0.41, 0.86)	1.11 (0.81, 1.53)	1.05 (0.77, 1.44)	1.23 (0.73, 2.08)
0.37 (0.17, 0.82)	0.95 (0.69, 1.30)	**0.34 (0.13, 0.92)**	**0.26 (0.11, 0.64)**	**0.29 (0.16, 0.55)**	Tianqi	**1.87 (1.15, 3.05)**	**1.77 (1.09, 2.87)**	**2.07 (1.09, 3.94)**
1.18 (0.73, 1.90)	**3.02 (1.26, 7.25)**	1.09 (0.51, 2.32)	0.84 (0.45, 1.55)	0.94 (0.47, 1.86)	**3.18 (1.26, 8.06)**	Jinqi	0.94 (0.77, 1.16)	1.11 (0.69, 1.77)
**1.37 (1.04, 1.80)**	**3.50 (1.60, 7.67)**	1.26 (0.75, 2.11)	0.97 (0.60, 1.57)	1.09 (0.62, 1.90)	**3.68 (1.58, 8.59)**	1.16 (0.67, 2.01)	Jinlida	1.17 (0.77, 1.79)
1.10 (0.56, 2.17)	**2.83 (1.04, 7.67)**	1.02 (0.73, 1.42)	0.79 (0.36, 1.72)	0.88 (0.38, 2.02)	**2.98 (1.04, 8.49)**	0.94 (0.41, 2.15)	0.81 (0.44, 1.50)	Tangmaikang

Note: Data of comparisons for the *Δ*LDL-C and *Δ*HDL-C are SMD (95% CI). The 95% confidence interval which does not range across 1 favors the column-defining treatment and is shown in bold.

#### 3.5.2 Secondary outcomes

**FIGURE 5:**
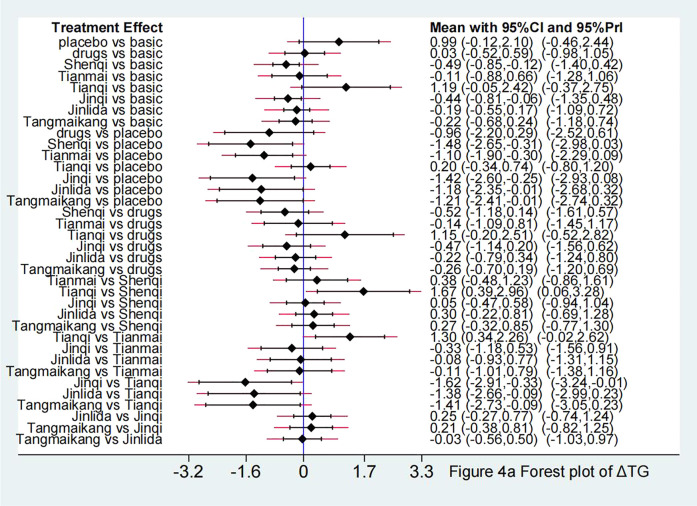
Forest plot in ΔTG.

**FIGURE 6:**
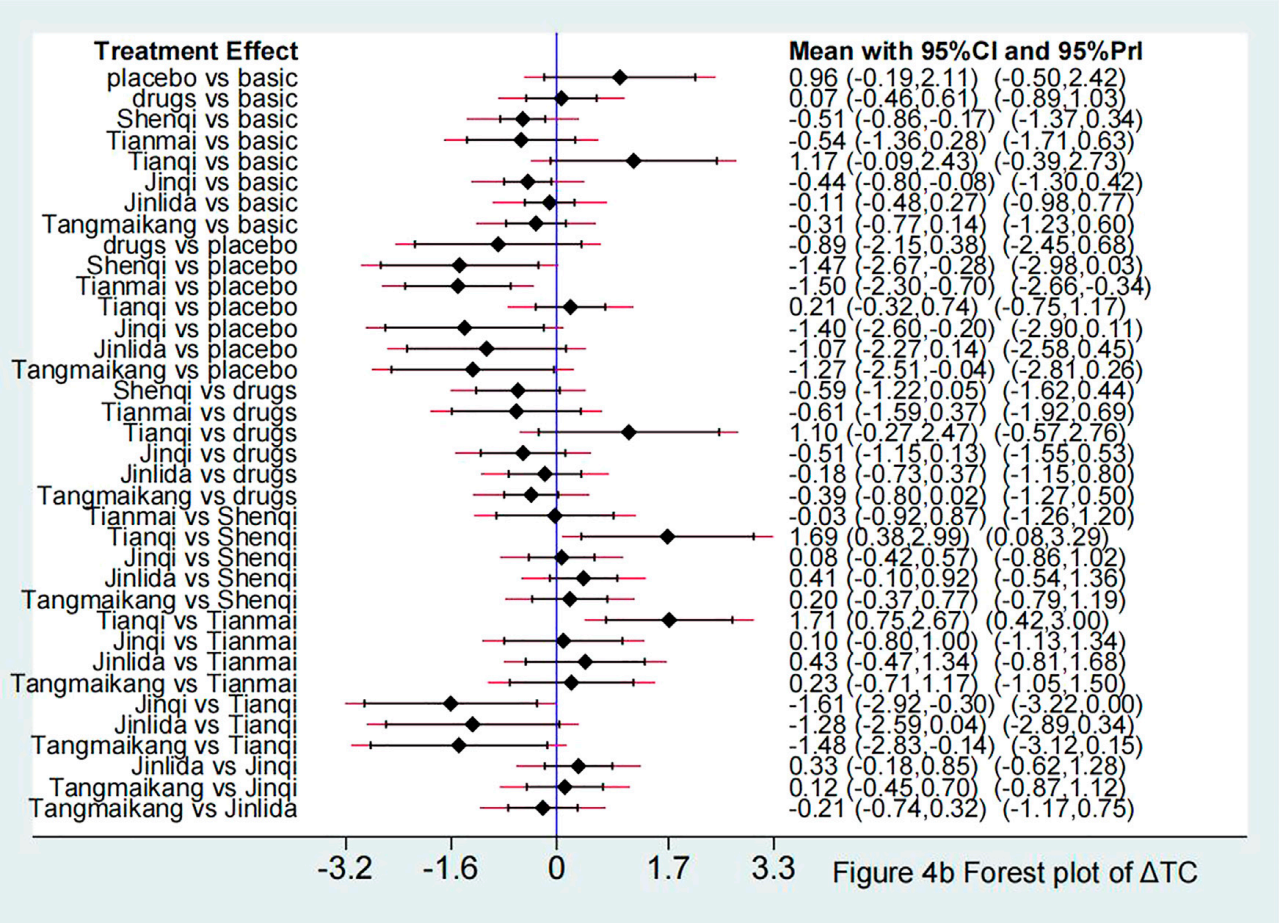
Forest plot in ΔTC.

Compared with LM, Shenqi + LM and Jinqi + LM showed statistically significant effect in lowering TG [Shenqi -0.49 (−0.85, −0.12), Jinqi -0.44 (−0.81, −0.06)] and TC [Shenqi −0.51 (−0.86, −0.17), Jinqi 0.44 (−0.80, −0.08)]. Compared with placebo + LM, except for Tianqi + LM [SMD 0.20 ( 95% CI: −0.34, 0.74)], all TCPM showed statistically significant effect in lowering TG, with SMD fluctuating between [−1.48 (95% CI: −2.65, −0.31)] (Shenqi + LM) to [−1.10 (95% CI: −1.90, −0.30)] (Tianmai + LM). Except for Tianqi + LM [SMD 0.21 (95%CI: −0.32, 0.74)] and Jinlida + LM [SMD −1.07 (95%CI: −2.27, 0.14)], other 4 TCPM showed statistically significant effect in lowering TC, with SMD fluctuating between [−1.50 (95%CI: −2.30, −0.70)] (Tianmai + LM) to [−1.27 (95%CI: −2.51, −0.04)] (Tangmaikang + LM). None of the six TCPMs showed a statistical difference in TG or TC compared with oral hypoglycaemic drugs + LM. Detailed comparative results are shown in Figures 5, 6 and Table 4B.

**TABLE 4B:** The league table of ΔTG and ΔTC.

Comparisons for ΔTG (bottom left) and ΔTC (upper right) of the 6 TPCM.								
basic	2.61 (0.83, 8.23)	1.08 (0.63, 1.84)	**0.60 (0.42, 0.84)**	0.58 (0.26, 1.33)	3.22 (0.91, 11.41)	**0.65 (0.45, 0.92)**	0.90 (0.62, 1.31)	0.73 (0.46, 1.15)
0.37 (0.12, 1.13)	placebo	0.41 (0.12, 1.46)	**0.23 (0.07, 0.76)**	**0.22 (0.10, 0.50)**	1.23 (0.73, 2.09)	**0.25 (0.07, 0.82)**	0.34 (0.10, 1.15)	**0.28 (0.08, 0.96)**
0.97 (0.56, 1.68)	2.60 (0.75, 8.99)	Oral drugs	0.56 (0.29, 1.05)	0.54 (0.20, 1.44)	3.00 (0.76, 11.82)	0.60 (0.32, 1.14)	0.84 (0.48, 1.45)	0.68 (0.45, 1.02)
**1.63 (1.13, 2.35)**	4.37 (1.36, 14.10)	1.68 (0.87, 3.27)	Shenqi	0.97 (0.40, 2.38)	**5.39 (1.46, 19.98)**	1.08 (0.66, 1.78)	1.51 (0.90, 2.51)	1.22 (0.69, 2.16)
1.12 (0.52, 2.42)	3.00 (1.35, 6.67)	1.16 (0.45, 2.99)	0.69 (0.29, 1.62)	Tianmai	**5.53 (2.12, 14.44)**	1.11 (0.45, 2.72)	1.54 (0.63, 3.82)	1.25 (0.49, 3.21)
0.30 (0.09, 1.05)	0.82 (0.48, 1.41)	0.32 (0.08, 1.22)	0.19 (0.05, 0.68)	0.27 (0.10, 0.72)	Tianqi	0.20 (0.05, 0.74)	0.28 (0.07, 1.04)	0.23 (0.06, 0.87)
**1.55 (1.06, 2.25)**	4.16 (1.29, 13.43)	1.60 (0.82, 3.12)	0.95 (0.56, 1.61)	1.38 (0.59, 3.27)	5.07 (1.40, 18.43)	Jinqi	1.40 (0.83, 2.33)	1.13 (0.63, 2.02)
1.21 (0.84, 1.73)	3.25 (1.01, 10.45)	1.25 (0.71, 2.20)	0.74 (0.44, 1.24)	1.08 (0.46, 2.54)	3.96 (1.10, 14.34)	0.78 (0.47, 1.31)	Jinlida	0.81 (0.48, 1.38)
1.25 (0.79, 1.98)	**3.35 (1.01, 11.17)**	1.29 (0.83, 2.02)	0.77 (0.43, 1.38)	1.12 (0.45, 2.75)	**4.09 (1.10, 15.29)**	0.81 (0.45, 1.46)	1.03 (0.61, 1.75)	Tangmaikang

Note: Data of comparisons for the ΔTG and ΔTC are SMD (95% CI). The 95% confidence interval which don’t range across 1 favors the column-defining treatment and are showed in bold.

**FIGURE 7:**
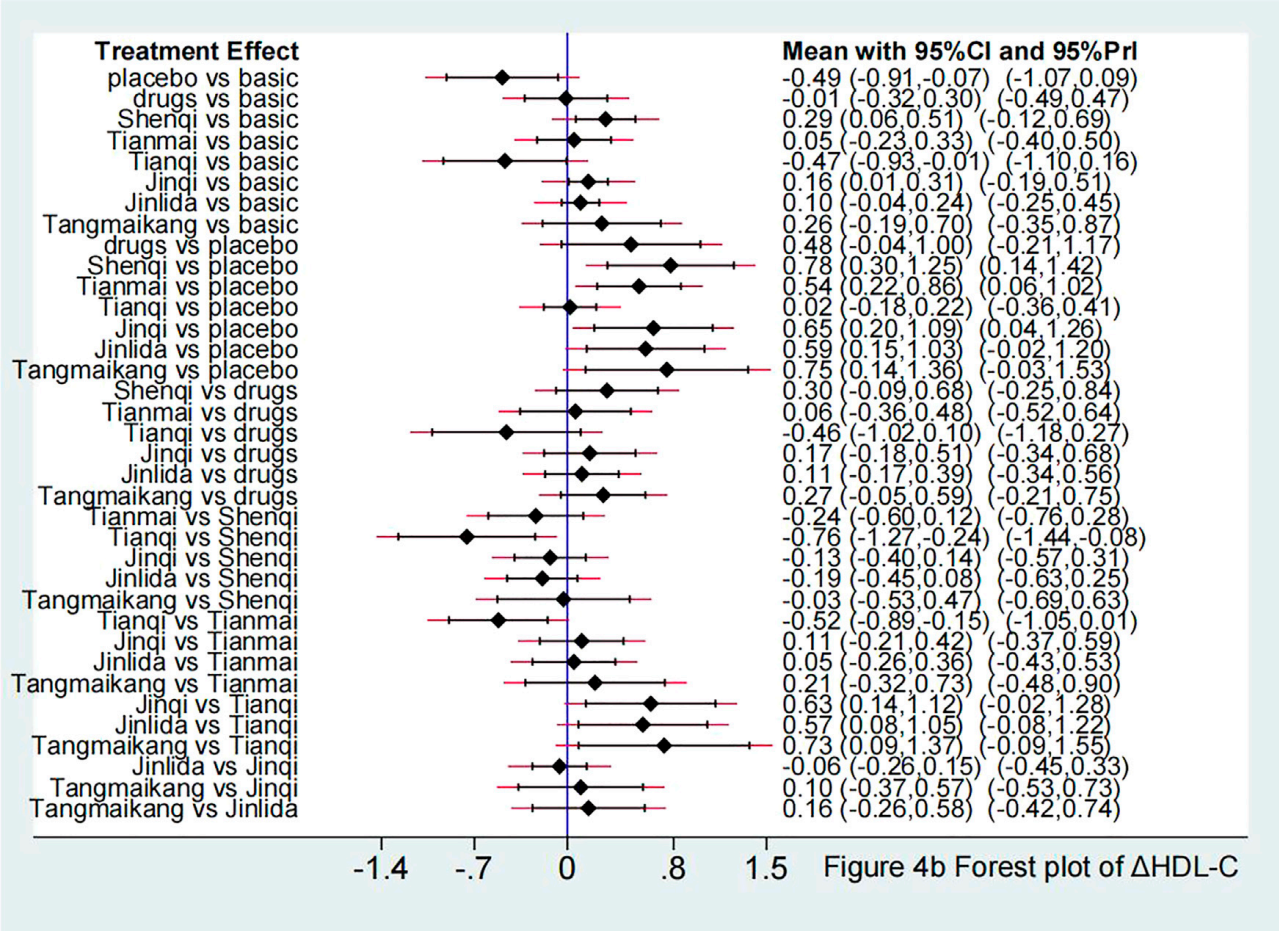
Forest plot in ΔHDL-C.

Compared with LM, Shenqi + LM [SMD 0.29 (95%CI: 0.06, 0.51)] and Jinqi + LM [SMD 0.16 (95%CI: 0.01, 0.31)] showed statistically significant effect in increasing HDL-C. Compared with placebo + LM, except for Tianqi + LM [SMD 0.02 (95%CI: −0.18, 0.22)], other 5 TCPM showed statistically significant effect in increasing HDL-C, with SMD fluctuating between [0.54 (95%CI: 0.22, 0.86)] (Tianmai + LM) to [0.78 (95%CI: 0.30, 1.25)] (Shenqi + LM). Detailed comparative results are shown in Figure 7 and Table 4A.

The treatment hierarchy was summarized and reported as the surface under the cumulative ranking curve (SUCRA) and mean ranks, which was shown in Supplementary File S5. The SUCRA plot of all TCPM in each outcome was shown in Supplementary File S6.

#### 3.5.3 Minimally contextualized framework

Considering all the crucial information and taking the simplicity and applicability across different contexts into account, the minimally contextualized framework was developed to draw a comprehensive conclusion regarding the effectiveness of interventions in the NMA. The LM was most closely connected to the other interventions and selected as the reference group, with an ineffective value, i.e., an absolute effect value of 0, as the decision threshold in outcomes. Interventions were classified as better, worse, and no different than the reference group based on whether the 95% confidence interval of the intervention vs. reference group effect size crossed the decision threshold. For all outcomes, the intervention has been divided into 2 categories, namely no different from the reference group (Category 0) and better or worse than the reference group (Category 1). Based on the same decision threshold, the interventions classified as more effective than the reference were compared against each other by examining whether the confidence or credible interval of their estimate of effect crosses the decision threshold. None intervention proved more effective than another Category 1 intervention, so there was no need to classify the intervention into a higher-level group. Then, the 8 interventions were separated into two main groups according to the certainty of the evidence, namely the group with high or moderate certainty evidence when compared with the reference, the and group with low or very low certainty evidence when compared with the reference. Last but not least, after examining the pairwise comparisons and ranking, those interventions ranked highest were ensured as among the most effective. As presented in Table 5.

**TABLE 5:** Final classification of 8 interventions for prediabetes.

Certainty of the evidence	Category	Intervention	Intervention vs.LM SMD (95% CI)	SUCRA
ΔLDL-C				
High certainty (moderate to high certainty evidence)	Category 1: among the most effective	None		
	Category 0: among the least effective	None		
Low certainty (low to very low certainty evidence)	Category 1: might be among the most effective	Jinlida + LM	−0.31 (−0.59, −0.04)	0.79
	Category 0: might be among the least effective	Shenqi + LM	−0.34 (−0.73, 0.05)	0.79
		Tianmai + LM	−0.23 (−0.72, 0.26)	0.68
		Jinqi + LM	−0.17 (−0.64, 0.31)	0.62
		Tangmaikang + LM	−0.10 (−0.78, 0.58)	0.56
		Oral drugs + LM	−0.08 (−0.67, 0.50)	0.53
		Placebo + LM	0.94 (0.21, 1.67)	0.09
		Tianqi + LM	0.99 (0.19, 1.79)	0.05
ΔTG				
High certainty (moderate to high certainty evidence)	Category 1: among the most effective	Shenqi + LM	−0.49 (−0.85, −0.12)	0.87
	Category 0: among the least effective	Jinlida + LM	−0.19 (−0.55, 0.17)	0.61
Low certainty (low to very low certainty evidence)	Category 1: might be among the most effective	Jinqi + LM	−0.44 (−0.81, −0.06)	0.83
	Category 0: might be among the least effective	Tangmaikang + LM	−0.22 (−0.68, 0.24)	0.65
		Tianmai + LM	−0.11 (−0.88, 0.66)	0.56
		Oral drugs + LM	0.03 (−0.52, 0.59)	0.41
		Placebo + LM	0.99 (−0.12, 2.10)	0.11
		Tianqi + LM	1.19 (−0.05, 2.42)	0.05
ΔTC				
High certainty (moderate to high certainty evidence)	Category 1: among the most effective	Shenqi + LM	−0.51 (−0.86, −0.17)	0.85
	Category 0: among the least effective	Jinlida + LM	−0.11 (−0.48, 0.27)	0.49
Low certainty (low to very low certainty evidence)	Category 1: might be among the most effective	Jinqi + LM	−0.44 (−0.80, -0.08)	0.78
		none		
	Category 0: might be among the least effective	Tianmai + LM	−0.54 (−1.36, 0.28)	0.80
		Tangmaikang + LM	−0.31 (−0.77, 0.14)	0.69
		Oral drugs + LM	0.07 (−0.46, 0.61)	0.35
		placebo + LM	0.96 (−0.19, 2.11)	0.13
		Tianqi + LM	1.17 (−0.09, 2.43)	0.05
ΔHDL-C				
High certainty (moderate to high certainty evidence)	Category 1: among the most effective	None		nN
	Category 0: among the least effective	Tianmai + LM	0.05 (−0.23, 0.33)	0.53
Low certainty (low to very low certainty evidence)	Category 1: might be among the most effective	Shenqi + LM	0.29 (0.06, 0.51)	0.89
		Jinqi + LM	0.16 (0.01, 0.31)	0.73
	Category 0: might be among the least effective	Tangmaikang + LM	0.26 (−0.19, 0.70)	0.81
		Jinlida + LM	0.10 (−0.04, 0.24)	0.61
		Oral drugs + LM	−0.01 (−0.32, 0.30)	0.41
		Tianqi + LM	−0.47 (−0.93, −0.01)	0.08
		Placebo + LM	−0.49 (−0.91, −0.07)	0.06

#### 3.5.4 Adverse reaction

Among the included studies, 10 reported adverse reactions. Eight reported gastrointestinal upset reactions in the treatment group (Zhou and Chen, 2003; Shen et al., 2006; Cao and Jin, 2010; Tan, 2010; Yan et al., 2011; Liu, 2015; Shi et al., 2016; Zhao, 2018), including diarrhea and bloating, and two reported no adverse reactions (Xiao et al., 2013; Yin, 2016). Six reported gastrointestinal upset reactions in the control group (Cao and Jin, 2010; Tan, 2010; Xiao et al., 2013; Liu, 2015; Shi et al., 2016; Yin, 2016), including nausea, vomiting, abdominal distention, and diarrhea. The adverse reactions were all mild and did not affect the treatment. No acute complications of diabetes such as hypoglycemia and ketoacidosis were reported, so the 6 TCPM could ameliorate dyslipidemia with a favorable safety profile.

#### 3.5.5 Network inconsistency and heterogeneity

There was no source of inconsistency for ΔLDL-C and ΔHDL-C. The global inconsistency test effect values (chi^2^) for ΔTG and ΔTC were 0.65 and 0.69 respectively, with no statistically significant difference (*p* ≥ 0.05). The local inconsistency test showed that there was no difference between each comparison (*p* ≥ 0.05). Therefore, no evidence of inconsistency existed in all networks. The detailed data of global and local inconsistency was presented in Supplementary File S7.

**FIGURE 8:**
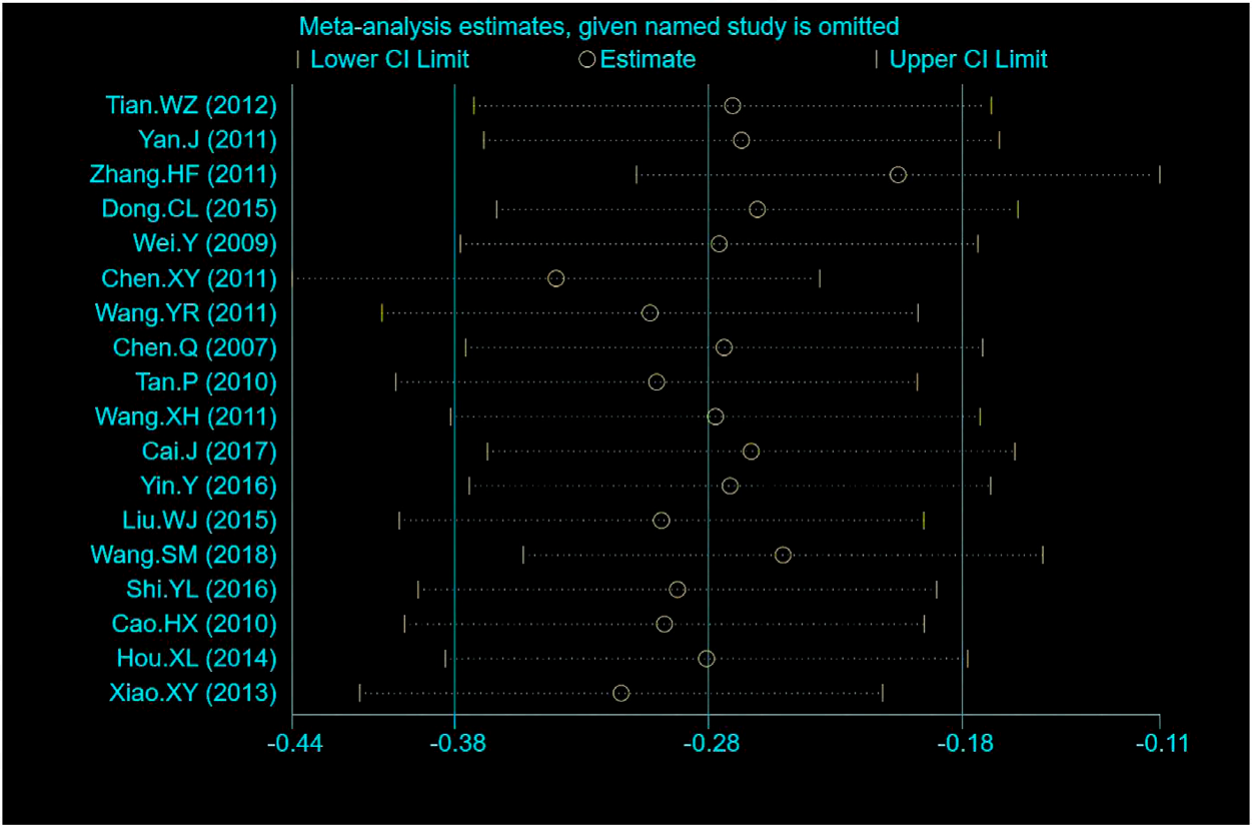
Sensitivity analysis of ΔLDL-C.

**FIGURE 9:**
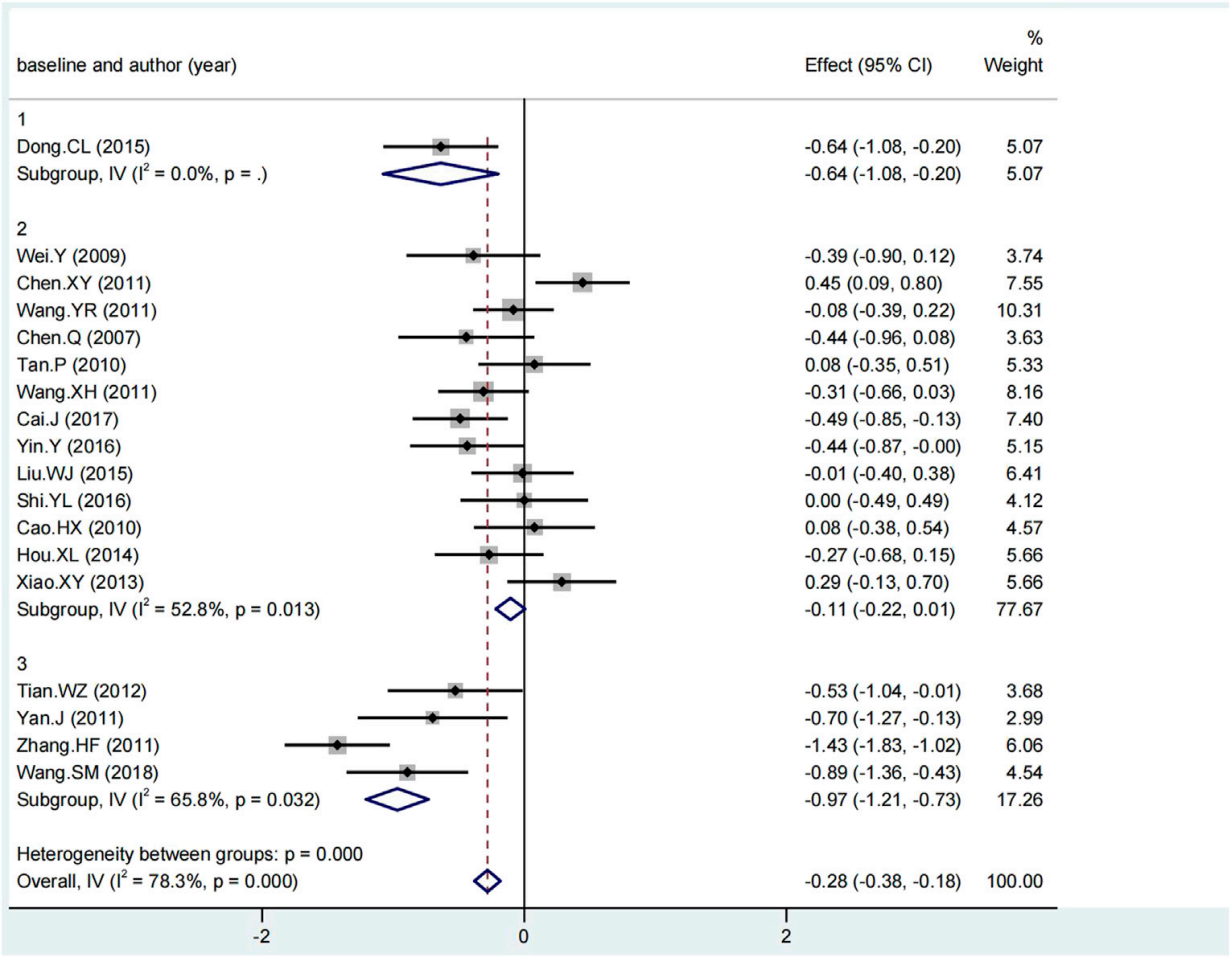
Subgroup analysis of ΔLDL-C.

The Chi-squared test and the I^2^ index for the primary outcome LDL-C indicated a high degree of heterogeneity among the included literature (I^2^ = 78.3%). For the comparisons with significant heterogeneity, we conducted sensitivity analysis as presented in Figure 8. Although the exclusion of Zhang HF (2011) would increase the upper CI limit by −0.19 and the exclusion of Wang YR (2011) would decrease the lower CI limit by −0.46, the exclusion from any one study did not exceed the expected confidence interval (−0.42, −0.25), suggesting the stability of meta-analysis. Meta-regression was performed to further investigate the sources of heterogeneity as presented in Table 6. The results indicated heterogeneity might stem from the baseline of LDL-C among these six factors (*p* = 0.076). Thus, subgroup analysis was conducted for the baseline of LDL-C as presented in Figure 9. One study reported the ideal LDL-C levels at baseline (˂2.6 mmol/L), 13 studies reported the appropriate LDL-C levels at baseline (2.6–3.4 mmol/L) and 4 studies reported pathologically elevated LDL-C levels (˃3.4 mmol/L). The heterogeneity analysis suggested that there was acceptable heterogeneity in subgroups (I^2^ = 0, 52.8%, 65.8%). The combined results of SMD (95% CI) in fixed-effect model analysis were respectively −0.64 (−1.08, −0.20), −0.11 (−0.22, 0.01) (*p* = 0.013) and −0.97 (−1.21, −0.73) (*p* = 0.032).

**TABLE 6:** Meta-regression of LDL-C.

_ES	Coef	Std. Err	t	P > t	lower CI	upper CI
Treatment duration	0.0657139	0.1628049	0.4	0.694	−0.2926173	0.4240452
Type of TCPM	0.0951485	0.0910892	1.04	0.319	−0.1053375	0.2956346
Control group	0.0392593	0.1306007	0.3	0.769	−0.2481908	0.3267094
Diagnostic criteria	−0.2053687	0.1292378	−1.59	0.14	−0.4898192	0.0790818
Risk of bias	−0.1367099	0.1656466	−0.83	0.427	−0.5012955	0.2278757
Baseline of LDL-C	−0.4039831	0.2059021	−1.96	0.076	−0.8571704	0.0492043
Cons	0.6724347	0.729448	0.92	0.376	−0.9330695	2.277939

Meta-regression: Number of obs = 18. REML estimate of between-study variance: tau^2^ = 0.1131. % residual variation due to heterogeneity: I-squared_res = 71.31%. Proportion of between-study variance explained: Adj R-squared = 29.52%. Joint test for all covariates: Model F(6, 11) = 1.8. With Knapp-Hartung modification: Prob > F = 0.1878.

#### 3.5.6 Publication bias

A funnel plot for risk of publication bias was shown in Supplementary File S8. The visual inspection of the funnel plot indicated that the nodes representing the comparison studies were evenly distributed at the ends of the null line. The fitted line was skewed at a greater angle, suggesting a small sample of events and a small possibility of publication bias for all outcomes.

## 4 Discussion

### 4.1 The heterogeneity in NMA

The test for inconsistency is a necessary process for NMA. It contains two major connotations of heterogeneity and consistency. Consistency refers to the agreement between direct and indirect comparisons and can be tested by a design-by-treatment approach or loop-specific approach. The heterogeneity refers to the difference between each pair of direct comparisons and includes clinical, methodological, and statistical differences. The I^2^ index gives the magnitude of statistical heterogeneity due to reasons other than the effect of chance (sampling error) (Higgins et al., 2019).

In this study, the consistency of direct and indirect comparisons was confirmed by the node-splitting analysis. Subsequently, the presence of non-negligible heterogeneity between included studies was confirmed in LDL-C. Meta-regression was thus performed for the different variables. Although none of the results were statistically different, the baseline values of LDL-C were the most likely source of heterogeneity, which was also consistent with the meta-regression of previous NMA (Zheng et al., 2020). The results of the subgroup analysis showed that the greater the baseline value, the more potent the lipid-lowering effect of TCPM if LDL-C did not reach the ideal value, which was consistent with previous results regarding the hypoglycaemic effect of TCPM (Jiang et al., 2021). Notably, among the 4 studies that reported pathologically elevated LDL-C levels, two studies using Shenqi (Tian WJ, Yan J) and one using jinlida (Wang SM). Both TCPMs were ranked highly in the NMA’s SUCRA, suggesting that the high ranking of the drugs in this NMA was associated with the high baseline of blood lipids. Therefore, the ranking result needs to be interpreted with caution when applied clinically.

### 4.2 Lipid monitoring and treatment are significant for the prevention of type 2 diabetes

Blood Lipids are the general term for lipids in blood plasma, including mainly total cholesterol (TC), triglycerides (TG), phospholipids, and free fatty acids. The level of lipoprotein cholesterol (LDL-C, HDL-C) reflects the level of lipoproteins (Kasper, 2016). Lipid monitoring and treatment are significant for the prevention of type 2 diabetes. On the one hand, prediabetes and diabetes are often associated with abnormalities in lipid metabolism. An analysis of the cross-sectional investigation of health and nutrition in the United States between 2011 and 2014 displayed that people with prediabetes (defined by ADA-FPG) were significantly more likely to have hyperlipidemia (51.2%) than the general population (Ali et al., 2018). On the other hand. Lipids are considered an essential hazard for the progression of prediabetes into diabetes and combined cardiovascular complications. A non-interventional cross-sectional study conducted in South Korea showed that prediabetes combined with abnormal lipid metabolism (high TG, low HDL-C) was 2.89 times more likely to develop cardiac complications later in life than patients with normal lipid metabolism (Kwon et al., 2021). Therefore, targeted measures should be taken to enhance the management of dyslipidemia in prediabetes.

Increased LDL-C is a major risk factor for the development of atherosclerosis (Jacobson et al., 2015). LDL enters the vascular wall through the endothelium and is modified to oxidized low-density lipoprotein (Ox-LDL) in the subendothelial layer. The latter grows and fuses to form the lipid core of the atherosclerotic plaque. In general, LDL-C is parallel to TC, but TC levels are also influenced by HDL-C levels, so LDL-C was recommended as an indicator of ASCVD risk in the guideline (Joint committee issued Chinese guideline for the management of dyslipidemia in adults, 2016). Therefore, LDL-C was chosen as the primary outcome of this study.

### 4.3 Shenqi granule was shown superior efficacy in TG and TC compared with other TCPM

The NMA analysis showed that no TCPM was among the most or least effective in reducing LDL-C based on the existing clinical studies. However, Shenqi + LM was among the most effective in reducing TG and TC, while Jinlida + LM was among the least effective.

Compared with other TCPM, Shenqi granule contains ginsenosides instead of *Panax ginseng* C.A.Mey. [Araliaceae], resulting in the richer active ingredient of ginseng in the formula. Roh et al. (2020) and Lu et al. (2020) have demonstrated that ginsenosides could improve lipid homeostasis in high-fat mice through various pathways, therefore significantly reducing body mass and serum levels of TC and TG. Meanwhile, pharmacological studies have shown that Shenqi granule could upregulate the expression of Bcl2 and relevant regulatory proteins and thus improve lipid metabolism (Zhang et al., 2019a). Network pharmacological prediction also found that Shenqi granule could participate in biological processes such as medium-density lipoprotein particle remodeling and RNA polymerase II promoter transcriptional regulation of glycolysis, leading to the reverse of the defective hepatic insulin signaling pathway and the improvement of lipid disorder (Shi et al., 2022). Thereby, Shenqi granule was shown superior efficacy in lowering TG and TC compared with other TCPM.

Although Jinlida also has been demonstrated its lipid-lowering effects in several preclinical studies (Table 3), the treatment duration of the clinical studies was far shorter than its *in vivo* experiments (10 weeks in Zhou HR and 15 weeks in Zhang H). In fact, it was also the shortest intervention time of the six TCPMs with an average intervention length of 3.5 weeks, which may explain its slightly weaker advantage of lowering lipids compared to other TCPMs.

### 4.4 Strengths and limitations

This study was to compare the differences in the efficacy of 6 TCPM based on the network meta-analysis. Its strength lies in the first exploration of the clinical use of different TCPMs to improve lipid profiles in prediabetic patients. Based on direct and indirect evidence, we provided a preliminary comprehensive ranking of these drugs in terms of their effects on lipids levels, which could provide a basis for future clinical research.

However, the study has the following potential limitations: ① the included studies were all conducted on the Chinese mainland without information on race and ethnicity, and the findings need to be extended with caution given the cross-racial and ethnic differences in prevalence and genetics of prediabetes. ② the period of treatment in the included literature varied widely, ranging from 1 month to 12 months ③ Most studies did not describe or qualify the specific means and duration of lifestyle modification, so potential sources of heterogeneity between studies may be related to the rigorousness and duration of lifestyle interventions. ④ Adverse events were poorly reported, the extent of adverse effects was not differentiated, and safety needs further confirmation. All of the aforementioned shortcomings may affect the authenticity of the results, so the ranking results should be viewed with caution and clinical application needs to be considered in the context of actual circumstances, expert opinion, and guidelines.

## 5 Conclusion

For patients with prediabetes, Traditional Chinese patent medicine Jinqi and Shenqi combined with lifestyle modification were associated with a significant reduction in TG and TC, while Shenqi + LM was among the most effective. Jinlida + LM was among the least effective. Jinqi + LM might be among the most effective in reducing TG and TC. However, head-to-head comparisons between drugs and further mechanistic exploration are warranted.

## Data Availability

The original contributions presented in the study are included in the article/Supplementary Material, further inquiries can be directed to the corresponding authors.
