# Supplementary material for: Treatment options of traditional Chinese patent medicines for dyslipidemia in patients with prediabetes: A systematic review and network meta-analysis
Source: Front Pharmacol. 2022 Aug 29;13:942563. doi: 10.3389/fphar.2022.942563 (PMC9465834; doi:10.3389/fphar.2022.942563)
Supplement: Supplementary file 1 [file DataSheet7.PDF]

Supplementary file 7a The global inconsistency

Outcome	chi^2	Prob > chi^2
ΔTG	0.65	0.4186
ΔTC	0.69	0.4050

Supplementary file 7b The local inconsistency

Outcome	side	Direct		Indirect		Difference			tau
		Coef.	Std. Err.	Coef.	Std. Err.	Coef.	Std. Err.	P>|z|	
ΔLDL-C	A E	-0.230	0.249	-1.170	49.969	0.940	49.969	0.985	0.237
	A H	-0.312	0.141	-0.230	50.199	-0.082	50.200	0.999	0.237
	B E	-1.170	0.280	-0.158	55.468	-1.012	55.469	0.985	0.237
	B F	0.052	0.160	-1.828	115.708	1.880	115.70	0.987	0.237
	C H	-0.230	0.263	-0.319	55.459	0.089	55.460	0.999	0.237
	C I	-0.018	0.171	0.147	115.516	-0.164	115.52	0.999	0.237
ΔTG	A E	-0.110	0.394	-1.100	50.008	0.990	50.009	0.984	0.388
	A H	-0.246	0.200	0.229	0.551	-0.474	0.586	0.419	0.397
	A I	-0.120	0.270	-0.594	0.521	0.474	0.586	0.419	0.397
	B E	-1.100	0.407	-0.034	55.470	-1.066	55.472	0.985	0.388
	B F	0.199	0.275	-1.781	115.453	1.980	115.45	0.986	0.388
	C H	0.000	0.404	-0.474	0.425	0.474	0.586	0.419	0.397
	C I	-0.349	0.260	0.126	0.526	-0.474	0.586	0.419	0.397
ΔTC	A E	-0.540	0.419	-1.500	50.008	0.960	50.010	0.985	0.365
	A H	-0.173	0.212	0.308	0.537	-0.481	0.578	0.405	0.378
	A I	-0.210	0.269	-0.690	0.512	0.481	0.578	0.405	0.378
	B E	-1.500	0.409	-0.466	55.466	-1.034	55.467	0.985	0.365
	B F	0.211	0.269	-1.709	115.362	1.920	115.36	0.987	0.365
	C H	0.050	0.400	-0.431	0.417	0.481	0.578	0.405	0.378
	C I	-0.468	0.236	0.013	0.526	-0.481	0.578	0.405	0.378
ΔHDL-C	A E	0.050	0.142	0.540	50.164	-0.490	50.164	0.992	0.133
	A H	0.099	0.072	0.110	67.103	-0.011	67.103	1.000	0.133
	B E	0.540	0.161	0.013	55.486	0.527	55.487	0.992	0.133
	B F	0.020	0.101	1.000	115.242	-0.980	115.24	0.993	0.133
	C H	0.110	0.141	0.097	89.359	0.013	89.359	1.000	0.133
	C I	0.270	0.163	0.290	196.191	-0.020	196.19	1.000	0.133
