# Supplementary material for: Treatment options of traditional Chinese patent medicines for dyslipidemia in patients with prediabetes: A systematic review and network meta-analysis
Source: Front Pharmacol. 2022 Aug 29;13:942563. doi: 10.3389/fphar.2022.942563 (PMC9465834; doi:10.3389/fphar.2022.942563)
Supplement: Supplementary file 2 [file DataSheet2.PDF]

Systematic review

Please select one of the options below to edit your record. Either option will create a new version of the record - the existing version will remain unchanged.

A list of fields that can be edited in an update can be found here

1. * Review title.

Give the title of the review in English

Comparative Efficacy of Traditional Chinese Patent Medicines Recommended from Guidelines for Blood Lipids in Patients with prediabetes: A network meta-analysis

2. Original language title.

For reviews in languages other than English, give the title in the original language. This will be displayed with the English language title.

基于指南推荐的六种中成药改善糖尿病前期血脂状况的网状 Meta 分析

3. * Anticipated or actual start date.

Give the date the systematic review started or is expected to start.

31/08/2021

4. * Anticipated completion date.

Give the date by which the review is expected to be completed.

17/10/2021

5. * Stage of review at time of this submission.

This field uses answers to initial screening questions. It cannot be edited until after registration.

Tick the boxes to show which review tasks have been started and which have been completed.

Update this field each time any amendments are made to a published record.

The review has not yet started: No

Review stage	Started	Completed
Preliminary searches	Yes	No
Piloting of the study selection process	Yes	No
Formal screening of search results against eligibility criteria	No	No
Data extraction	No	No
Risk of bias (quality) assessment	No	No
Data analysis	No	No

Provide any other relevant information about the stage of the review here.

6. * Named contact.

The named contact is the guarantor for the accuracy of the information in the register record. This may be any member of the review team.

Li Jiang

Email salutation (e.g. "Dr Smith" or "Joanne") for correspondence:
Mr Jiang

7. * Named contact email.

Give the electronic email address of the named contact.

294143396@qq.com

8. Named contact address

PLEASE NOTE this information will be published in the PROSPERO record so please do not enter private information, i.e. personal home address

Give the full institutional/organisational postal address for the named contact.

11 road, Chaoyang District, Beijing University of Chinese Medicine, Beijing, China

9. Named contact phone number.

Give the telephone number for the named contact, including international dialling code.

18811701807

10. * Organisational affiliation of the review.

Full title of the organisational affiliations for this review and website address if available. This field may be completed as 'None' if the review is not affiliated to any organisation.

Dongzhimen Hospital affiliated to Beijing University of Chinese Medicine, Beijing, China

Organisation web address:

11. * Review team members and their organisational affiliations.

Give the personal details and the organisational affiliations of each member of the review team. Affiliation refers to groups or organisations to which review team members belong.
NOTE: email and country now MUST be entered for each person, unless you are amending a published record.

Mr Li Jiang. Dongzhimen Hospital affiliated to Beijing University of Chinese Medicine, Beijing, China
Zhuang Li. Dongzhimen Hospital affiliated to Beijing University of Chinese Medicine, Beijing, China
Mr Taiqi Xue. Dongzhimen Hospital affiliated to Beijing University of Chinese Medicine, Beijing, China

12. * Funding sources/sponsors.

Details of the individuals, organizations, groups, companies or other legal entities who have funded or sponsored the review.

This work was supported by grants from The General Project of National Natural Science Foundation of China, Project for Young Teachers of Beijing University of Chinese Medicine and National Key R&D Program of China and the Project of Beijing Science and Technology Subject

Grant number(s)

State the funder, grant or award number and the date of award

No. 81774272, No.2019-JYB-JS-080，No：2018YFC1704400，No：2018YFC1704402，Z191100008319004

13. * Conflicts of interest.

List actual or perceived conflicts of interest (financial or academic).

None

14. Collaborators.

Give the name and affiliation of any individuals or organisations who are working on the review but who are not listed as review team members. **NOTE: email and country must be completed for each person, unless you are amending a published record.**

15. * Review question.

State the review question(s) clearly and precisely. It may be appropriate to break very broad questions down into a series of related more specific questions. Questions may be framed or refined using PI(E)COS or similar where relevant.

The aim of this meta-analysis of randomized controlled trials is to evaluate the efficacy and safety of adjunctive Traditional Chinese Patent Medicines recommended from guidelines for blood lipids in patients with prediabetes.

16. * Searches.

State the sources that will be searched (e.g. Medline). Give the search dates, and any restrictions (e.g. language or publication date). Do NOT enter the full search strategy (it may be provided as a link or attachment below.)

PubMed, Web of Science, The Cochrane Library, EMbase, CNKI, WanFang and VIP databases were searched by computer. The retrieval time was from the establishment of each database to July 1, 2021. According to the specific situation of each database, the combination of subject words and free words was used to ensure the comprehensiveness of the retrieval.

Pre-specified search keywords were “Prediabetes”，“impaired glucose tolerance”，“impaired fasting glucose”，” Chinese patent medicine”，” Chinese traditional patent medicine”，“Shenqi”，“Tianmai”，“Tianqi”，“Jinqi”，“Jinlida”，“Tangmaikang” and “randomized controlled trial”.

17. URL to search strategy.

Upload a file with your search strategy, or an example of a search strategy for a specific database, (including the keywords) in pdf or word format. In doing so you are consenting to the file being made publicly accessible.

Or provide a URL or link to the strategy. Do NOT provide links to your search **results**.

Do not make this file publicly available until the review is complete

18. * Condition or domain being studied.

Give a short description of the disease, condition or healthcare domain being studied in your systematic review.

Prediabetes is a state of abnormal glucose metabolism between normal blood glucose and diabetes, including impaired fasting glucose (IFG) or impaired glucose tolerance (IGT) or both (IFG+IGT). Dyslipidemia is a high risk factor to increase the incidence of cardiovascular complications in diabetes mellitus, and an independent risk factor to induce coronary atherosclerosis. Clinical studies have shown that Traditional Chinese Patent Medicines had the unique superiority in improving blood lipid status for prediabetes and relieving symptoms. Domestic and western guidelines for prediabetes have clearly recommended several kinds of Traditional Chinese Patent Medicines. Although there were meta-analyses on the clinical efficacy of Traditional Chinese Patent Medicines in the treatment of prediabetes, the following shortcomings still existed: (1) The main focus is on blood glucose indicators, such as Hba1c, FBG and PBG, and there was a lack of sufficient attention to blood lipids. (2) The

number of included literatures was small, the sample size was insufficient, and the heterogeneity among different comparisons was large; (3) Most studies only reported the direct comparison between Chinese patent medicine and western medicine or placebo, and there was still a lack of efficacy comparison between various types of Chinese patent medicines.

19. * Participants/population.

Specify the participants or populations being studied in the review. The preferred format includes details of both inclusion and exclusion criteria.

inclusion criteria : (1) types of studies: randomized controlled trials (RCTs); (2) type of participants: patients diagnosed with prediabetes. (3) Main intervention: The control group was treated with oral hypoglycemic drugs or LM, while the experimental group was treated with LM combined with CTPM, including Shen qi jiang tang capsule/granule/tablet, Tian mai xiao ke capsule/granule/table, Tian qi capsule/ granule/tablet, Jin qi jiang tang capsule/granule/table, Jin li da capsule/granule/table, Tang mai kang capsule/granule/tablet. (4) Intervention time of clinical studies that included HbA1c was longer than 12 weeks to meet the base time for the change of HbA1c. (5) Outcomes: blood lipids, including triglyceride(TG)、total cholesterol (TC)、low-density lipoprotein cholesterol(LDL-C)、high-density lipoprotein cholesterol(HDL-C)

exclusion criteria: (1) The intervention of the experimental group was decoctions, herbal extracts, or other synthetic plant-based drugs; (2) In the experimental group, the intervention was combined with oral hypoglycemic drugs or other Chinese herbal medicines or appropriate technology of TCM, such as acupuncture, point application therapy and massage; (3) Unknown dosage of intervention drugs and unmatched composition of prescription; (4) Studies with incomplete general data and baseline indicators. (5) Observational studies, repeated publications and studies without full text.

20. * Intervention(s), exposure(s).

Give full and clear descriptions or definitions of the interventions or the exposures to be reviewed. The preferred format includes details of both inclusion and exclusion criteria.

The experimental group was treated with LM combined with CTPM, including Shen qi jiang tang capsule/granule/tablet, Tian mai xiao ke capsule/granule/table, Tian qi capsule/ granule/tablet, Jin qi jiang tang capsule/granule/table, Jin li dacapsule/granule/table, Tang mai kang capsule/granule/tablet. The authors defined TCPM as refined dosage forms, such as capsules, tablets, oral liquid, and powders, which originated from herbal medicine (whole plants or their adjuncts), relative standardization in composing the main effective components, and manufactured in accordance with the PRC Pharmacopoeia’ s monograph.

21. * Comparator(s)/control.

Where relevant, give details of the alternatives against which the intervention/exposure will be compared (e.g. another intervention or a non-exposed control group). The preferred format includes details of both inclusion and exclusion criteria.

The control group was treated with oral hypoglycemic drugs or LM. The two groups maintained the same baseline in basic characteristics, routine treatment measures (health education, diet control, antihypertensive drugs, lipid-lowering drugs), course of treatment, and related indicators before treatment.

22. * Types of study to be included.

Give details of the study designs (e.g. RCT) that are eligible for inclusion in the review. The preferred format includes both inclusion and exclusion criteria. If there are no restrictions on the types of study, this should be stated.

Randomized clinical trials will be included irrespective of blinding, publication status or language

23. Context.

Give summary details of the setting or other relevant characteristics, which help define the inclusion or exclusion criteria.

24. * Main outcome(s).

Give the pre-specified main (most important) outcomes of the review, including details of how the outcome is defined and measured and when these measurement are made, if these are part of the review inclusion criteria.

triglyceride(TG)、total cholesterol (TC)、low-density lipoprotein cholesterol(LDL-C)、high-density lipoprotein cholesterol(HDL-C)

Measures of effect
SMD

25. * Additional outcome(s).

List the pre-specified additional outcomes of the review, with a similar level of detail to that required for main outcomes. Where there are no additional outcomes please state 'None' or 'Not applicable' as appropriate to the review

Not applicable

Measures of effect

26. * Data extraction (selection and coding).

Describe how studies will be selected for inclusion. State what data will be extracted or obtained. State how this will be done and recorded.

Two researchers independently screened the literature with reference to the Cochrane Collaboration Systematic Evaluators manual (version 5.1.0). The titles and abstractions of the literature were reviewed by EndNote X9. After initial elimination of irrelevant and duplicate literature, the full text was obtained and read. The included literatures were cross-checked, and differences were discussed or judged by a third researcher. Data extraction contents: demographic characteristics, diagnostic criteria, randomized method, allocation plan, treatment and control measures, drug dosage, drug composition, trial period, clinical efficacy and biochemical indicators

27. * Risk of bias (quality) assessment.

State which characteristics of the studies will be assessed and/or any formal risk of bias/quality assessment tools that will be used.

The literature quality assessment followed the risk of bias assessment tool of the Cochrane manual and was completed by two investigators using Software Review Manager 5.3，The tool standard covers 7 aspects: ①evaluation of randomization; ②allocation concealment; ③blinding of participants and personnel; ⑤blinding of outcome assessment; ⑥selective reporting; ⑦other biases. For each study, the above 7 items were evaluated as "low bias", "high bias" and "unclear" (lack of relevant information or uncertain bias).The differences were reviewed by the third member, and finally determined and drew the bias risk map after discussion.

28. * Strategy for data synthesis.

Describe the methods you plan to use to synthesise data. This **must not be generic text** but should be **specific to your review** and describe how the proposed approach will be applied to your data.
If meta-analysis is planned, describe the models to be used, methods to explore statistical heterogeneity, and software package to be used.

Software Review Manager 5.3 was used to directly compare the various treatments in primary outcomes. The forest plot was applied to represent the heterogeneity of the combination of treatments. The network meta-analysis was based on the frequency model. The statistical analysis and drawing of network diagram were performed by using software STATA 15.0 with reference to Lin Xia, et al [15], The study effect sizes were synthesized by using a random-effects NMA model. Analysis package in STATA 15.0 included metan, mvmeta, metareg and so on. The normal likelihood was used to perform all analyses of continuous outcomes, and the binomial likelihood(assuming a normal distribution) logic link model was used for the analysis of dichotomous outcomes. the network evidence map was drawn to show the comparison between the interventions. The need for loop inconsistency detection was accordingly determined by whether there was a closed loop between the interventions. The design-by-treatment approach was used to check the consistency in the entire NMA. Standardized mean difference (SMD) was used for measurement data and odds ratio (OR) was used for counting data. Each effect size was represented by 95% confidence interval(CI). If the 95% CI of OR did not cross the effect line 1, and the 95% CI of SMD did not cross the effect line 0, P value<0.05, indicating that the difference was statistically significant. The value of surface under the cumulative ranking (SUCRA) of various interventions was calculated. Accordingly the ranking of efficacy among interventions were represented in a single diagram. Last but not least, the correction funnel plot was developed to identify evidence for small sample effects.

29. * Analysis of subgroups or subsets.

State any planned investigation of 'subgroups'. Be clear and specific about which type of study or participant will be included in each group or covariate investigated. State the planned analytic approach.

Six types of Traditional Chinese Patent Medicine were analyzed as subgroups. Several studies of Each type were be combined respectively. The heterogeneity and statistical effect value of each subgroup were calculated. Sensitivity analysis and meta-regression were performed when the heterogeneity was too large to ensure that the heterogeneity was less than 50%

30. * Type and method of review.

Select the type of review, review method and health area from the lists below.

Type of review

Cost effectiveness	No
Diagnostic	No
Epidemiologic	No
Individual patient data (IPD) meta-analysis	No
Intervention	Yes
Living systematic review	No
Meta-analysis	No
Methodology	No
Narrative synthesis	No
Network meta-analysis	Yes
Pre-clinical	No
Prevention	No
Prognostic	No
Prospective meta-analysis (PMA)	No
Review of reviews	No
Service delivery	No
Synthesis of qualitative studies	No
Systematic review	Yes
Other	No

Health area of the review

Alcohol/substance misuse/abuse	No
Blood and immune system	No

Cancer	No
Cardiovascular	No
Care of the elderly	No
Child health	No
Complementary therapies	Yes
COVID-19	No
Crime and justice	No
Dental	No
Digestive system	No
Ear, nose and throat	No
Education	No
Endocrine and metabolic disorders	Yes
Eye disorders	No
General interest	No
Genetics	No
Health inequalities/health equity	No
Infections and infestations	No
International development	No
Mental health and behavioural conditions	No
Musculoskeletal	No
Neurological	No
Nursing	No
Obstetrics and gynaecology	No
Oral health	No
Palliative care	No
Perioperative care	No
Physiotherapy	No
Pregnancy and childbirth	No
Public health (including social determinants of health)	No

Rehabilitation	No
Respiratory disorders	No
Service delivery	No
Skin disorders	No
Social care	No
Surgery	No
Tropical Medicine	No
Urological	No
Wounds, injuries and accidents	No
Violence and abuse	No

31. Language.

Select each language individually to add it to the list below, use the bin icon to remove any added in error.

English

There is an English language summary.

32. * Country.

Select the country in which the review is being carried out. For multi-national collaborations select all the countries involved.

China

33. Other registration details.

Name any other organisation where the systematic review title or protocol is registered (e.g. Campbell, or The Joanna Briggs Institute) together with any unique identification number assigned by them.
If extracted data will be stored and made available through a repository such as the Systematic Review Data Repository (SRDR), details and a link should be included here. If none, leave blank.

34. Reference and/or URL for published protocol.

If the protocol for this review is published provide details (authors, title and journal details, preferably in Vancouver format)

No I do not make this file publicly available until the review is complete

35. Dissemination plans.

Do you intend to publish the review on completion?

Yes

36. Keywords.

Give words or phrases that best describe the review. Separate keywords with a semicolon or new line. Keywords help PROSPERO users find your review (keywords do not appear in the public record but are included in searches). Be as specific and precise as possible. Avoid acronyms and abbreviations unless these are in wide use.

Traditional Chinese patent medicine; Guidelines; prediabetes; Blood lipids; network meta-analysis

37. Details of any existing review of the same topic by the same authors.

If you are registering an update of an existing review give details of the earlier versions and include a full bibliographic reference, if available.

38. * Current review status.

Update review status when the review is completed and when it is published.
New registrations must be ongoing so this field is not editable for initial submission.

Review_Ongoing

39. Any additional information.

Provide any other information relevant to the registration of this review.

40. Details of final report/publication(s) or preprints if available.

Leave empty until publication details are available OR you have a link to a preprint (NOTE: this field is not editable for initial submission).
List authors, title and journal details preferably in Vancouver format.
