# Supplementary material for: Treatment options of traditional Chinese patent medicines for dyslipidemia in patients with prediabetes: A systematic review and network meta-analysis
Source: Front Pharmacol. 2022 Aug 29;13:942563. doi: 10.3389/fphar.2022.942563 (PMC9465834; doi:10.3389/fphar.2022.942563)
Supplement: Supplementary file 3 [file DataSheet4.PDF]

Supplementary file 4-GRADE assessment

		Certainty assessment							№ of patients			
Intervention of studies	Study design	Risk of bias	Inconsistency	Indirectness	Imprecision	Publication bias	Intransivity	Incoherence	Treatment group	Control group	Certainty	Importance
ΔLDL-C (follow up: 3-24 months)												CRITICAL
Shenqi+LM vs LM	randomized trials	serious[1]	not serious	not serious	serious[4]	none	not serious	not serious	55	55	LOW	
Tianmai+LM vs LM	randomized trials	serious1	not serious	serious[3]	serious[4]	none	not serious	not serious	42	42	VERY LOW	
Tianmai+LM vs placebo+LM	randomized trials	serious[1]	not serious	serious[3]	serious[4]	none	not serious	not serious	60	60	VERY LOW	
Tianqi+LM vs placebo+LM	randomized trials	serious[1]	not serious	not serious	serious[4]	none	not serious	serious[6]	183	163	VERY LOW	
Jinqi+LM vs LM	randomized trials	serious[1]	not serious	not serious	serious[4]	none	not serious	not serious	74	69	LOW	
Jinlida+LM vs metformin+LM	randomized trials	very serious[1]	not serious	not serious	not serious	suspected[5]	not serious	not serious	42	41	VERY LOW	
Jinlida+LM vs LM	randomized trials	very serious[1]	serious[2]	not serious	not serious	suspected[5]	not serious	not serious	253	242	VERY LOW	
Tangmaikang+LM vs metformin+LM	randomized trials	very serious[1]	not serious	not serious	serious[4]	none	not serious	not serious	126	126	VERY LOW	
ΔTC (follow up: mean 1-24 months)/TG (follow up: 1-24 months)												IMPORTANT
Shenqi+LM vs LM	randomized trials	serious[1]	not serious	not serious	not serious	none	not serious	not serious	175	151	MODERATE	
Tianmai+LM vs LM	randomized trials	serious[1]	not serious	serious[3]	not serious	suspected[5]	not serious	not serious	42	42	LOW	

Tianmai+LM vs placebo+LM	randomized trials	serious[1]	not serious	serious[3]	serious[4]	none	not serious	not serious	60	60	VERY LOW	
Tianqi+LM vs placebo+LM	randomized trials	very serious[1]	not serious	not serious	not serious	none	not serious	not serious	183	163	VERY LOW	
Jinqi+LM vs LM	randomized trials	serious[1]	not serious	serious[3]	not serious	none	not serious	not serious	168	171	LOW	
Jinlida+LM vs metformin+LM	randomized trials	serious[1]	not serious	not serious	not serious	none	not serious	not serious	42	41	MODERA TE	
Jinlida+LM vs LM	randomized trials	very serious[1]	not serious	serious[3]	serious[4]	none	not serious	serious[6]	253	242	VERY LOW	
Tangmaikang+LM vs LM	randomized trials	serious[1]	not serious	serious[3]	serious[4]	none	not serious	not serious	90	92	VERY LOW	
Tangmaikang+LM vs metformin+LM	randomized trials	serious[1]	not serious	serious[3]	serious[4]	none	not serious	not serious	126	126	VERY LOW	
ΔHDL-C(follow up: mean 1-24 months)												IMPORTANT
Shenqi+LM vs LM	randomized trials	serious[1]	serious[2]	not serious	not serious	none	not serious	not serious	55	55	LOW	
Tianmai+LM vs LM	randomized trials	serious[1]	not serious	not serious	not serious	none	not serious	not serious	42	42	MODERA TE	
Tianmai+LM vs placebo+LM	randomized trials	serious[1]	not serious	serious[3]	serious[4]	none	not serious	not serious	60	60	VERY LOW	
Tianqi+LM vs placebo+LM	randomized trials	serious[1]	not serious	serious[3]	not serious	none	not serious	not serious	183	163	LOW	
Jinqi+LM vs LM	randomized trials	serious[1]	not serious	serious[3]	not serious	none	not serious	not serious	138	139	LOW	
Jinlida+LM vs	randomized	serious[1]	not serious	not serious	serious[4]	suspected[5]	not serious	not serious	42	41	VERY	

metformin+LM	trials										LOW
Jinlida+LM vs LM	randomized trials	very serious[1]	not serious	not serious	serious[4]	none	not serious	serious[6]	211	205	VERY LOW
Tangmaikang+LM vs metformin+LM	randomized trials	serious[1]	not serious	not serious	not serious	suspected[5]	not serious	serious[6]	36	36	VERY LOW

1 There were serious or very serious limitations in the included randomized trials according to the Rob scale (mostly for the lack of binding and allocation concealment)

2 There was a high level of heterogeneity between the included studies

3 The comparisons included few studies and no available information about effect modifiers

4 The 95% confidence interval included no effect and the upper or lower confidence limit crossed the minimal important difference

5 Too few published papers and every paper reported a positive result of interest

6 The results of the direct and indirect comparisons were judged to be serious in incoherence by comparing the basic characteristics of the included RCTs.
