# Supplementary material for: Treatment options of traditional Chinese patent medicines for dyslipidemia in patients with prediabetes: A systematic review and network meta-analysis
Source: Front Pharmacol. 2022 Aug 29;13:942563. doi: 10.3389/fphar.2022.942563 (PMC9465834; doi:10.3389/fphar.2022.942563)
Supplement: Supplementary file 4 [file DataSheet6.PDF]

Supplemental file 6 SUCRA comparative figure

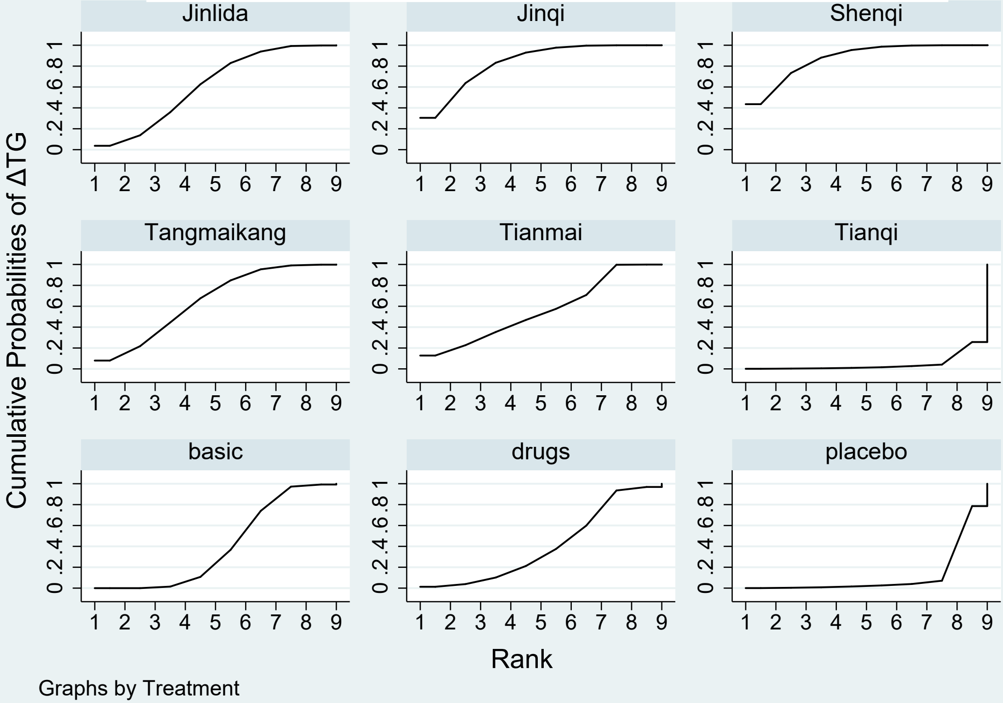

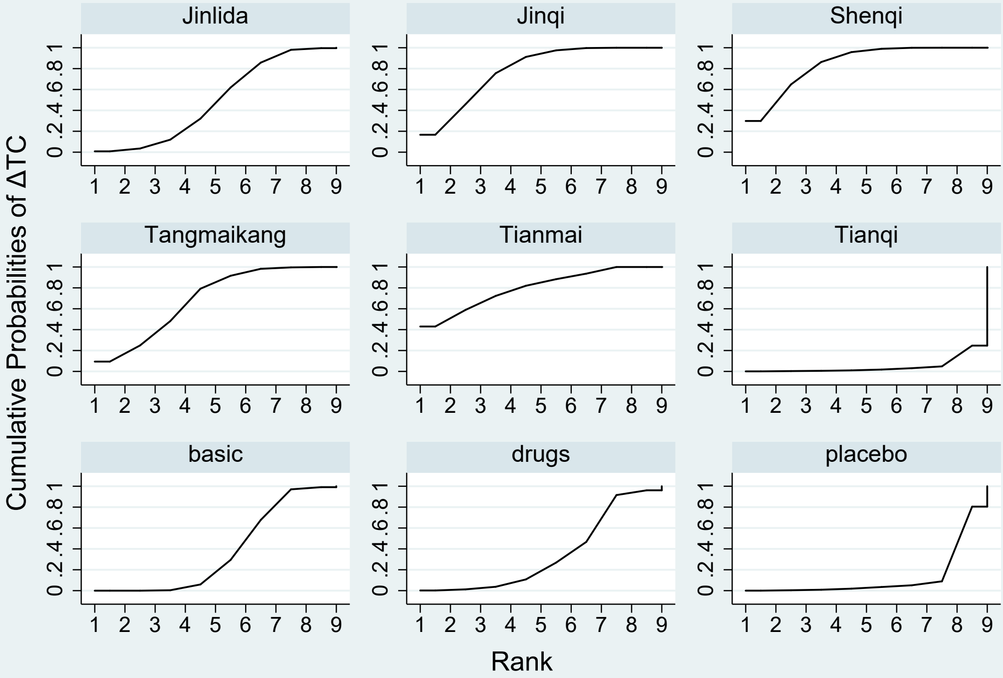

Graphs by Treatment

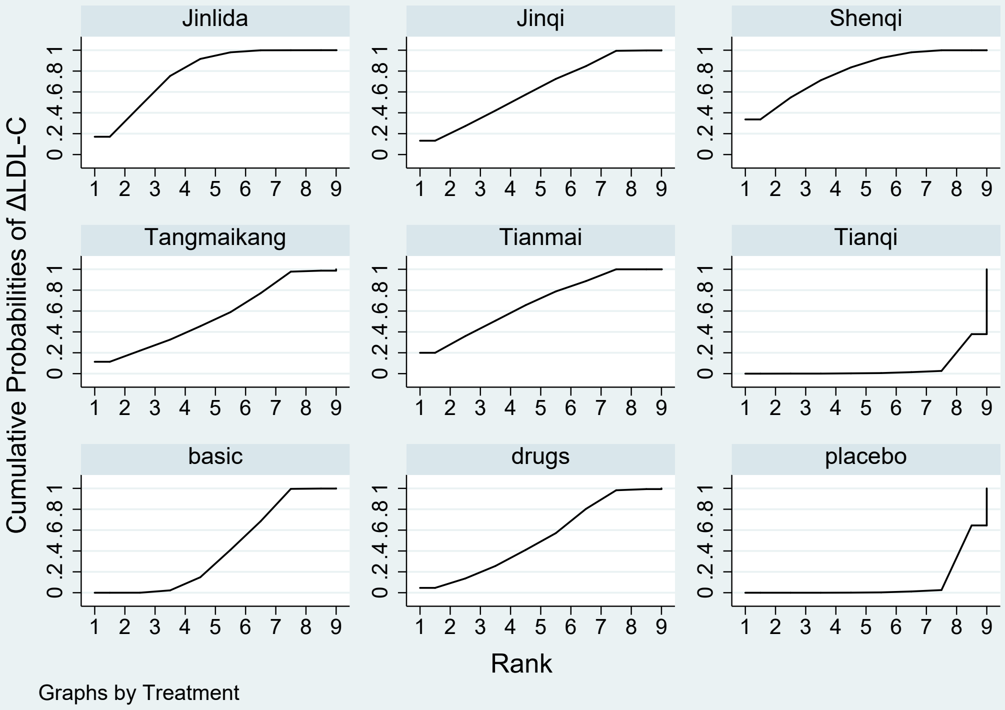
Jinlida
Jinqi
Shenqi
Tangmaikang
Tianmai
Tianqi
basic
drugs
placebo
Cumulative Probabilities of ΔLDL-C
Rank
0 .2 .4 .6 .8 1
1 2 3 4 5 6 7 8 9
Graphs by Treatment

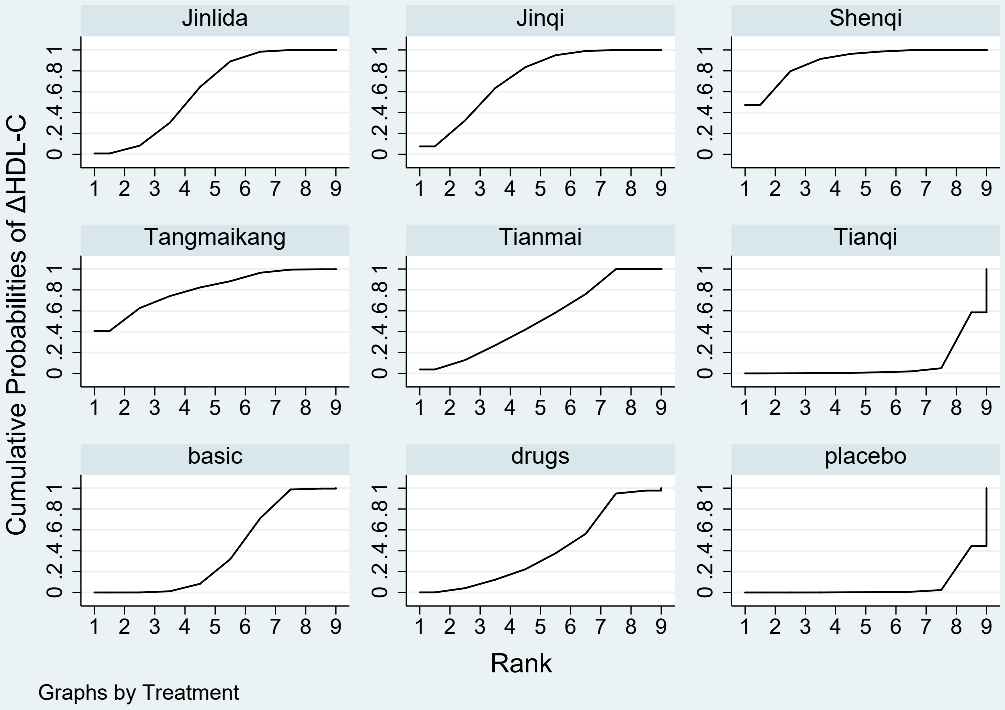

Jinlida
Jinqi
Shenqi
Tangmaikang
Tianmai
Tianqi
basic
drugs
placebo
0 .2 .4 .6 .8 1
1 2 3 4 5 6 7 8 9
Cumulative Probabilities of ΔHDL-C
Rank
Graphs by Treatment
