# Supplementary material for: Treatment options of traditional Chinese patent medicines for dyslipidemia in patients with prediabetes: A systematic review and network meta-analysis
Source: Front Pharmacol. 2022 Aug 29;13:942563. doi: 10.3389/fphar.2022.942563 (PMC9465834; doi:10.3389/fphar.2022.942563)
Supplement: Supplementary file 5 [file Table1.DOCX]

**Table 1** Characteristics of the included studies

| Study ID  (Author+time) | Sample size  (M/F) | Age (year) | Diagnostic  criteria | Intervention |  | Duration  (month) | Outcome  measure |
| --- | --- | --- | --- | --- | --- | --- | --- |
|  |  |  |  | Treatment group | Control group |  |  |
| Chen C 2005 (25) | T:46(22/14) C:32(18/14) | T:53.1±10.1 C:52.5±9.3 | WHO 1999 | Shenqi capsule(0.7g tid)+LM | LM | 12 | ①② |
| Lin JH 2007 (26) | T:29(unclear) C:29(unclear) | T:53.6±4.4 C:52.9±5.8 | WHO 1999 | Shenqi granule(3g bid)+LM | LM | 6 | ①② |
| Tian WZ 2012 (27) | T:30(17/13) C:30(15/15) | T:50.6±6.4 C:51.2±6.2 | WHO 1999 | Shenqi granule(3g tid)+LM | LM | 6 | ①②③④ |
| Yan J 2011 (28) | T:25(13/12) C:25(14/11) | T:51.4±2.2 C:50.6±2.4 | ADA 2008 | Shenqi granule(3g bid)+LM | LM | 3 | ①②③④ |
| Zhao Q 2018 (29) | T:45(13/32) C:45(15/30) | T:51.5±4.8 C:51.3±4.3 | ADA 2010 | Shenqi granule(3g tid)+LM | LM | 3 | ①② |
| Dong CL 2015 (30) | T:42(18/24) C:42(20/22) | T:52.4±8.6 C:54.1±7.9 | CDS 2013 | Tianmai tablet(0.24g bid)+LM | LM | 6 | ①②③④ |
| Zhang HF 2011 (31) | T:60(28/32) C:60(29/31) | - | WHO 1999 | Tianmai tablet(0.24g bid)+LM | LM+placebo | 24 | ①②③④ |
| Wei Y 2009 (32) | T:30(10/19) C:30(10/21) | T:52.6±6.10 C:51.7±5.6 | WHO 1999 | Tianqi capsule(8g tid)+LM | LM+placebo | 6 | ①②③④ |
| Chen XY 2011 (33) | T:63(27/36) C:59(32/27) | T:52.8±10.5 C:52.9±10.9 | WHO 1999 | Tianqi capsule(8g tid)+LM | LM+placebo | 12 | ①②③④ |
| Wang YR 2011 (34) | T:90(40/54) C:74(34/40) | T:51.4±8.7 C:51.7±9.3 | WHO 1999 | Tianqi capsule(8g tid)+LM | LM+placebo | 12 | ①②③④ |
| Chen Q 2007 (35) | T:32(18/14) C:27(15/12) | T:44±8.6 C:45±9.1 | ADA 1997 | Jinqi tablet(2.52g tid)+LM | LM | 1 | ①②③④ |
| Mao LH 2003 (36) | T:32(15/17) C:30(14/16) | T:64.8±5.4 C:63.9±5.8 | WHO 1985 | Jinqi tablet(2.94g tid)+LM | LM | 3 | ①② |
| Tan P 2010 (37) | T:42(20/22) C:42(21/21) | T:58.4±2.1 C:54.8±1.6 | ADA 2008 | Jinqi tablet(3.36g tid)+LM | LM | 3 | ①②③④ |
| Zhou DY 2003 (38) | T:46(18/28) C:42(17/25) | T:55.6±12.1 C:54.0±11.3 | WHO 1985 | Jinqi tablet(2.94g tid)+LM | LM | 12 | ①②④ |
| Zhou ZN 2002 (39) | T:24(9/15) C:22(8/14) | T:45.4±7.0 C:46.0±6.8 | WHO 1985 | Jinqi tablet(4.2g tid)+LM | LM | 1 | ①②④ |
| Wang XH 2011 (40) | T:65(35/30) C:65(33/32) | T:72.6±4.1 C:73.1±3.8 | WHO 1999 | Jinlida granule(9g tid)+LM | LM | 6 | ①②③④ |
| Cai J 2017 (41) | T:60(32/28) C:60(30/30) | T:46.4±10.6 C:48.2±9.6 | CDS 2013 | Jinlida granule(9g tid)+LM | LM | 3 | ①②③④ |
| Liu WJ 2015 (42) | T:52(24/28) C:49(22/27) | T:49.6±11.3 C:47.9±11.8 | WHO 1999 | Jinlida granule(9g tid)+LM | LM | 3 | ①②③④ |
| Wang SM 2018 (43) | T:42(23/19) C:37(20/17) | T:57.1±10.6 C:55.6±11.4 | CDS 2010 | Jinlida granule(9g tid)+LM | LM | 4 | ①②③ |
| Yin Y 2016 (44) | T:42(22/20) C:41(23/18) | T:47.8±7.1 C:48.4±6.8 | CDS 2010 | Jinlida granule(9g tid)+LM | LM+metformin | 2 | ①②③④ |
| Shi YL 2016 (45) | T:32(17/15) C:29(14/15) | T:47.1±7.1 C:49.9±7.2 | WHO 1999 | Jinlida granule(9g tid)+LM | LM | 3 | ①②③④ |
| Gu JW 2007 (46) | T:36(unclear) C:36(unclear) | 42.3 | WHO 1999 | Tangmaikang granule(5g tid)+LM | LM | 24 | ①② |
| Shen YD 2006 (47) | T:27(unclear) C:28(unclear) | 55.4±8.7 | ADA 2003 | Tangmaikang granule(5g bid)+LM | LM | 3 | ①② |
| Tao LW 2012 (48) | T:27(14/13) C:28(9/19) | T:55.1±10.4 C:55.4±7.7 | ADA 2003 | Tangmaikang granule(5g bid)+LM | LM | 3 | ①② |
| Cao HX 2010 (49) | T:36(22/14) C:36(20/16) | T:55.5±2.6 C:55.4±2.4 | ADA 2008 | Tangmaikang granule(5g tid)+LM | LM+metformin | 3 | ①②③④ |
| Hou XL 2014 (50) | T:45(26/19) C:45(28/17) | T:67.5±5.8 C:65.4±6.2 | WHO 1999 | Tangmaikang granule(5g tid)+LM | LM+metformin | 3 | ①②③ |
| Xiao XY 2013 (51) | T:45(27/18) C:45(26/19) | - | ADA 2006 | Tangmaikang granule(5g tid)+LM | LM+metformin | 3 | ①② |
| Note:T：Treatment group；C：Control group；LM：lifestyle modification；①TC([Total](D:/translator/Dict/8.9.9.0/resultui/html/index.html#/javascript:;) [cholesterol](D:/translator/Dict/8.9.9.0/resultui/html/index.html#/javascript:;))；②TG(triglyceride)；③LDL- C；④HDL- C | | | | | | | |
