# Supplementary material for: Treatment options of traditional Chinese patent medicines for dyslipidemia in patients with prediabetes: A systematic review and network meta-analysis
Source: Front Pharmacol. 2022 Aug 29;13:942563. doi: 10.3389/fphar.2022.942563 (PMC9465834; doi:10.3389/fphar.2022.942563)
Supplement: Supplementary file 6 [file DataSheet3.PDF]

Supplemental File 3 Search strategy in PubMed

search strategy	[PubMed]
Prediabetes	#1 “Prediabetic States”[Mesh] OR “State, Prediabetic*”[All Fields] OR “States, Prediabetic*”[All Fields] OR “prediabetes*”[All Fields] OR “impaired glucose tolerance*”[All Fields] OR “Glucose Intolerances*” OR “Intolerance, Glucose*”[All Fields] OR “Intolerances, Glucose ”[Mesh]OR“Impaired Glucose Tolerance*”[All Fields]OR“Glucose Tolerance, Impaired”[All Fields]OR “Glucose Tolerances, Impaired”[All Fields]OR“Impaired Glucose Tolerances*”[All Fields]OR“Tolerance, Impaired Glucose*”[All Fields]or “Tolerances, Impaired Glucose*”[All Fields]OR“Impaired Glucose Regulation*”[All Fields]OR“impaired fasting glucose*”[All Fields]
Intervention	#2 “Drugs, Chinese Herbal” [Mesh] OR “Chinese Drugs, Plant” [All Fields] OR “Chinese Herbal Drugs” [All Fields] OR “Herbal Drugs, Chinese” [All Fields] OR “Plant Extracts, Chinese” [All Fields] OR “Chinese Plant Extracts” [All Fields] OR “Extracts, Chinese Plant” [All Fields]<br>#3 “Chinese patent medicine”[All Fields] OR “Chinese patent medicine injection”[All Fields] OR “Traditional Chinese Patent Medicine ”[All Fields] OR “modern traditional chinese patent medicine[All Fields] OR “oral chinese patent medicine”[All Fields] OR “Chinese patent drugs”[All Fields] OR “Chinese Traditional Patent Medicine”[All Fields]<br>#4 “Shenqijiangtang Granules”[All Fields] OR “Shenqijiangtang Capsule”[All Fields] OR “Shenqijiangtang Tablets”[All Fields] OR“Shenqi”[All Fields]OR “Jinqijiangtang Granules”[All Fields]OR “Jinqijiangtang Capsule”[All Fields]OR “Jinqijiangtang Tablets”[All Fields]OR“Jinqi”[All Fields] OR“Jinlida Granules”[All Fields] OR “Jinlida Capsule”[All Fields] OR “Jinlida Tablets”[All Fields] OR“Jinlida”[All Fields]<br>#5 “Tianmai Granules”[All Fields] OR “Tianmai Capsule”[All Fields] OR “Tianmai Tablets”[All Fields] OR“Tianmai”[All Fields]OR “Tianqi Granules”[All Fields]OR “Tianqi Capsule”[All Fields]OR “Tianqi Tablets”[All Fields]OR“Tianqi”[All Fields] OR“Tangmaikang Granules”[All Fields] OR “Tangmaikang Capsule”[All Fields] OR “Tangmaikang Tablets”[All Fields] OR“Tangmaikang”[All Fields]
Intervention	#6 “Treatment*”[All Fields] OR Therapy[All Fields] OR Therapies[All Fields]
Outcome	#7 “Dyslipidemias” [Mesh] OR “Dyslipidemia” [All Fields] OR “Dyslipoproteinemias” [All Fields] OR “Dyslipoproteinemia” [All Fields] OR “Hyperlipemia” [All Fields] OR “Hyperlipemias” [All Fields] OR “Hyperlipidemia” [All Fields]OR “Lipidemia” [All Fields] OR “Lipidemias” [All Fields] OR “Lipemia” [All Fields]OR “Lipemias” [All Fields] OR “Hypercholesterolemia” [All Fields] OR “Hyperlipoproteinemias” [All Fields] OR “Hypertriglyceridemia” [All

	Fields]
Study	#8 “randomized controlled trial”[pt] OR “controlled clinical trial”[pt] OR” randomized”[tiab] OR randomised[tiab] OR randomly[tiab] OR randomization[tiab] OR randomisation[tiab] OR random allocation[mh] OR placebo[mh] OR placebo[tiab] OR trial[tiab] OR groups[tiab]
Final Search	#9 #1AND (#2 OR #3 OR #4 OR #5 OR #6 OR #7) AND #8

Clinical studies published before July 2022 were retrieved by combining subject words with free words and linking corresponding Boolean logical operators.

Web of Science

#1 TS=(Prediabetic States) OR TS=(State, Prediabetic) OR TS=(States, Prediabetic) OR TS=(prediabetes) OR TS=(State, Prediabetic) OR TS=(impaired glucose tolerance) OR TS=(Glucose Intolerances) OR TS=(Intolerance, Glucose) OR TS=(Intolerances, Glucose) OR TS=(Impaired Glucose Tolerance) OR TS=(Glucose Tolerance, Impaired) OR TS=(Glucose Tolerances, Impaired) OR TS=(Impaired Glucose Tolerances)OR TS=(Tolerance, Impaired Glucose) OR TS=(Tolerances, Impaired Glucose) OR TS=(Impaired Glucose Regulation) OR TS=(impaired fasting glucose)

#2 TS=(Chinese patent medicine) OR AB=(Chinese patent medicine injection) OR AB=(Traditional Chinese Patent Medicine) OR AB=(modern traditional chinese patent medicine)OR AB=(oral chinese patent medicine) OR AB=(Chinese patent drugs) OR AB=(Chinese Traditional Patent Medicine)

#3 TS=(Clinical Trial) OR KP=(Adaptive Clinical Trial) OR KP=(Clinical Trial, Phase I) OR KP=(Clinical Trial, Phase II) OR KP=(Clinical Trial, Phase II) OR KP=(Clinical Trial, Phase III) OR KP=(Clinical Trial, Phase IV) OR KP=(Controlled Clinical Trial) OR KP=(Randomized Controlled Trial)

#4 #1 AND #2 AND #3

Cochrane Library

#1 MeSH descriptor: [Prediabetic State] this term only

#2 Prediabetic State* or Impaired Glucose Tolerance* or impaired fasting glucose* or Impaired Glucose Regulation*:ti,ab,kw

#3 #1 or #2

#4 MeSH descriptor: [Medicine, Chinese Traditional] this term only

#5 Chinese patent medicine* or Traditional Chinese Patent Medicine* or oral Chinese patent medicine* or Chinese patent drug* or Chinese Traditional Patent Medicine*:ti,ab,kw

#6 #4 or #5

#7 MeSH descriptor: [Dyslipidemias] this term only

#8 Dyslipoproteinemia* or Hyperlipemia* or Lipidemia* or Hypercholesterolemia* or Hypertriglyceridemia*:ti,ab,kw

#9 #7 or #8

#10 #3 and #6

#11 #9 and #6

#12 #10 or #11

Embase

#1 'impaired glucose tolerance'/exp OR 'chemical diabetes' OR 'chemical diabetes mellitus':ti,ab,kw OR 'diabetes mellitus, potential':ti,ab,kw OR 'diabetes, chemical':ti,ab,kw OR 'diabetes, latent':ti,ab,kw OR 'chemical diabetes mellitus':ti,ab,kw OR 'genetic prediabetes':ti,ab,kw OR 'chemical diabetes mellitus':ti,ab,kw OR 'chemical diabetes mellitus':ti,ab,kw OR 'glucose tolerance impairment':ti,ab,kw OR 'glucose tolerance, potentially impaired':ti,ab,kw OR 'impaired glucose tolerance, potential':ti,ab,kw OR 'latent diabetes mellitus':ti,ab,kw OR 'potential diabetes':ti,ab,kw OR 'potential glucose tolerance impairment':ti,ab,kw OR 'pre diabetes mellitus':ti,ab,kw OR 'prediabetes':ti,ab,kw OR 'prediabetes mellitus':ti,ab,kw OR 'prediabetic stage':ti,ab,kw OR 'prediabetic stage':ti,ab,kw OR 'prediabetic state':ti,ab,kw

#2 'Chinese medicinal formula'/exp OR 'chinese medicine'/exp OR 'fang ji fen lei':ti,ab,kw OR 'traditional Chinese medicinal formula':ti,ab,kw OR 'traditional Chinese medicinal formulas':ti,ab,kw OR 'chinese patent medicine':ti,ab,kw OR 'chinese patent drug':ti,ab,kw OR 'chinese traditional patent medicine':ti,ab,kw OR 'oral chinese patent medicine':ti,ab,kw OR 'shenqi jiangtang':ti,ab,kw OR 'shenqi':ti,ab,kw OR 'jinqi jiangtang':ti,ab,kw OR 'jinqi':ti,ab,kw OR 'tianmai xiaoke':ti,ab,kw OR 'tianmai':ti,ab,kw OR 'jinlida':ti,ab,kw OR 'tianqi':ti,ab,kw OR 'tangmaikang':ti,ab,kw

#3 'dyslipidemia'/exp OR 'dyslipaemia':ti,ab,kw OR 'dyslipemia':ti,ab,kw OR 'dyslipidaemia':ti,ab,kw OR 'dyslipidaemias':ti,ab,kw OR 'dyslipidemias':ti,ab,kw OR 'lipidaemia, dys':ti,ab,kw OR 'lipidemia, dys':ti,ab,kw

#4 'randomized controlled trial'/exp OR 'controlled trial, randomized':ti,ab,kw OR 'randomised controlled study':ti,ab,kw OR 'randomised controlled trial':ti,ab,kw OR 'randomized controlled study':ti,ab,kw OR 'trial, randomized controlled':ti,ab,kw

#5 1# AND #2 AND #3 AND #4

ClinicalTrial.gov

prediabetic stage | Interventional Studies | Impaired Glucose Tolerance | Chinese Medicinal Formulation

Search strategy in Chinese database

Search	Query
#1	‘‘impaired glucose tolerance’’[mh] or IGT[mh] or ‘‘abnormal glucose intolerance’’[mh] or hyperglycemia[mh] or ‘‘prediabetes’’[mh] or ‘‘pre-diabetes’’[mh]
#2	‘‘traditional Chinese medicine’’[mh] or ‘‘Chinese herb*’’[mh] or ‘‘traditional Chinese patent medicine’’[mh] or ‘‘Chinese herbal medicine’’[mh] or ‘‘plant extract’’[tiab] or ‘‘capsule’’[mh] or ‘‘granule’’[mh] or ‘‘tablet’’[mh] or ‘‘pill’’[mh] or ‘‘Shenqi jiangtang capsule’’[tiab] or“Shen qi jiang tang granule”[tiab] or “Shen qi” [tiab] or “Tian mai xiao ke tablet”’[tiab] or “Tianmai” [tiab] or ‘‘Tianqi capsule’’ [tiab] or “Tianqi jiangtang capsule” [tiab] or “Tianqi” [tiab] or “Jin qi jiang tang tablet”[tiab] or “Jinqi”[tiab] or “Jinlida capsule”[tiab] or “Jinlida”[tiab] or ‘‘Tang mai kang capsule’’[tiab] or ‘‘Tangmaikang”[tiab]
#3	‘‘Blood lipids’’[mh] or ‘‘Triglycerides’’[mh] or ‘‘Triglyceride’’[mh] or ‘‘Total

	cholesterol''[mh] or ''Lipoproteins''[tiab] or ''Low-density lipoproteins''[mh] or ''High-density lipoproteins''[mh]
#4	randomized trial[tiab] or clinical observation[tiab]
#5	#1 AND #2 AND #3 AND #4 AND #5
