# Supplementary material for: Treatment options of traditional Chinese patent medicines for dyslipidemia in patients with prediabetes: A systematic review and network meta-analysis
Source: Front Pharmacol. 2022 Aug 29;13:942563. doi: 10.3389/fphar.2022.942563 (PMC9465834; doi:10.3389/fphar.2022.942563)
Supplement: Supplementary file 7 [file Table2.DOCX]

**Table 2** Patented formulations of the Included TPCM

| Study | Formulation | Source | Species,  concentration | Quality control reported?  (Y/N) | Chemical analysis  reported? (Y/N) |
| --- | --- | --- | --- | --- | --- |
| Chen C (25) Lin JH et al (26) Tian WZ et al(27) Yan J et al (28) Zhao Q (29) | Shenqi capsule/granule | [Henan Lingrui Pharmaceutical, Co. Ltd.]  SFDA approval number: Z10970002 | 1.Total Ginsenoside of *Panax ginseng* C.A.Mey.[Araliaceae] from stems and leaves (Renshen jing ye zaogan) 6g,  2.*Astragalus mongholicus* Bunge [Fabaceae] (Huangqi), 124g,  3.*Dioscorea oppositifolia* L. [Dioscoreaceae](Shanyao), 62g,  4.*Poria cocos* (Schw. ) Wolf.(Fuling), 62g  5.*Rehmannia glutinosa* (Gaertn.) DC. [Orobanchaceae](Dihuang), 186g  6.*Ophiopogon japonicus* (Thunb.) Ker Gawl. [Asparagaceae](Maidong), 62g  7.*Schisandra chinensis* (Turcz.) Baill. [Schisandraceae](Wuweizi), 62g  8.*Trichosanthes kirilowii* Maxim. [Cucurbitaceae](Tianhuafen), 62g  9.*Rubus chingii* Hu [Rosaceae](Fupenzi), 31g  10.*Alisma plantago-aquatica subsp. orientale* (Sam.) Sam. [Alismataceae](Zexie), 62g  11.*Lycium chinense* Mill. [Solanaceae](Gouqi), 124g  Made into 1000 capsules | Y-Prepared  according to Chinese Pharmacopoeia (2020 Edition) (52) | Y – HPLC (53), UPLC-MS/MS (54), RP-HPLC (55), UPLC-Q-TOF MS (56), GC-MS (57) |
| Dong CL et al  (30)  Zhang HF et al (31) | Tianmai tablet | [Hebei Fuge Pharmaceutical, Co. Ltd.]  SFDA approval number: Z20049007 | 1.Chromium picolinate,1.6mg  2.*Trichosanthes kirilowii* Maxim. [Cucurbitaceae](Tianhuafen), unknown dosage  3.*Schisandra chinensis* (Turcz.) Baill. [Schisandraceae](Wuweizi), unknown dosage  4.*Ophiopogon japonicus* (Thunb.) Ker Gawl. [Asparagaceae](Maidong)  Unknown dosage | N | N |
| Wei Y (32)  Chen XY (33)  Wang YR et al (34) | Tianqi capsule | [Heilongjiang Weimingtianren Pharmaceutical, Co. Ltd.]  SFDA approval number: Z20063799 | 1.*Panax ginseng* C.A.Mey.[Araliaceae]  2.*Astragalus mongholicus* Bunge [Fabaceae] (Huangqi)  3.*Trichosanthes kirilowii* Maxim. [Cucurbitaceae](Tianhuafen)  4.*Ligustrum lucidum* W.T.Aiton.[Oleaceae](Nvzhenzi),  5*.Eclipta prostrata* (L.) L.[Asteraceae](Hanliancao),  6.*Coptis chinensis* Franch.[Ranunculaceae](Huanglian),  7.*Dendrobium nobile* Lindl.[Orchidaceae] (Shihu),  8.*Lycium chinense* Mill.[Solanaceae](Digupi),  9.*Rhus chinensis* Mill.[Anacardiaceae](Wubeizi),  10.*Cornus officinalis* Siebold & Zucc. [Cornaceae](Shanzhuyu)  Unknown dosage | Y-Prepared  according to the State Drug Administration standard WS-666 (Z-186) 2002, TLCS was used to determine the content of berberine hydrochloride in Huanglian and TLC was used to identify the thin layer chromatography of Nvzhenzi,Renshen,Huangqi, Huanglian and Wubeizi (58) | Y – UPLC-LTQ Orbitrap HRMS (59) |
| Chen Q et al (35)  Mao LH (36)  Tan P (37)  Zhou DY et al(38)  Zhou ZN (39) | Jinqi tablet | [Tianjin Zhongxing Pharmaceutical, Co. Ltd.]  SFDA approval number: Z10920027 | 1.*Astragalus mongholicus* Bunge [Fabaceae] (Huangqi), 513g  2.*Coptis chinensis* Franch. [Ranunculaceae](Huanglian), 343g  3.*Lonicera japonica* Thunb. [Caprifoliaceae](Jinyinhua), 2058g  Pressed into 1000 tablets | Y-Prepared  according to Chinese Pharmacopoeia (2020 Edition) | Y –LC-MS/MS (60),  UPLC-ESI-MS (61) |
| Wang XH et al (40)  Cai J et al (41)  Liu WJ (42)  Wang SM et al (43)  Yin Y (44)  Shi YL et al (45) | Jinlida granule | [Shijiazhuang Yiling  Pharmaceutical, Co. Ltd.]  SFDA approval number: Z20050845 | 1.*Panax ginseng* C.A.Mey.[Araliaceae](Renshen), 184.5g  2.*Polygonatum sibiricum* Redouté [Asparagaceae](Huangjing), 244.5g  3.*Atractylodes lancea* (Thunb.) DC. [Asteraceae](Chao Cangzhu), 122.2g  4.*Sophora flavescens* Aiton [Fabaceae](Kushen) 100g  5.*Ophiopogon japonicus* (Thunb.) Ker Gawl. [Asparagaceae](Maidong), 244.5g  6.*Rehmannia glutinosa* (Gaertn.) DC[Orobanchaceae](Dihuang),184.5g  7.Reynoutria multiflora (Thunb.) Moldenke [Polygonaceae](Heshouwu) 149g  8. *Cornus officinalis* Siebold & Zucc. [Cornaceae](Shanzhuyu), 244.5g  9.*Poria cocos* (Schw. ) Wolf.(Fuling), 149g  10.Eupatorium fortunei Turcz. [Asteraceae](Peilan), 100g  11.*Coptis chinensis* Franch. [Ranunculaceae](Huanglian), 100g  12.*Anemarrhena asphodeloides* Bunge [Asparagaceae](Zhimu), 122.2g  13.*Epimedium sagittatum* (Siebold & Zucc.) Maxim. [Berberidaceae]  (Yinyanghuo), 100g   1. *Salvia miltiorrhiza* Bunge [Lamiaceae](Danshen), 160g   15.*Pueraria montana var.lobata* (Willd.) Maesen & S.M.Almeida ex Sanjappa & Predeep [Fabaceae](Gegen), 244.5g  16.*Litchi chinensis* Sonn. [Sapindaceae](Lizhihe), 244.5g  17.*Lycium chinense* Mill. [Solanaceae](Digupi), 149g  Ethanol extracted and concentrated to 1000g, 9g per granule | Y-Prepared  according to Chinese Pharmacopoeia (2020 Edition) | Y –HPLC (62),  UV Spectrophotometry (63) |
| Gu JW (46)  Shen YD et al(47)  Tao LW et al (48)  Cao HX et al (49)  Hou XL et al (50)  Xiao XY et al (51) | Tangmaikang capsule | [Sichuan Shenghe  Pharmaceutical, Co. Ltd.]  SFDA approval number: Z20090557 | 1.*Astragalus mongholicus* Bunge [Fabaceae] (Huangqi), 200g  2.*Rehmannia glutinosa* (Gaertn.) DC. [Orobanchaceae](Dihuang), 216.7g  3.*Paeonia lactiflora* Pall. [Paeoniaceae](Chishao), 216.7g  4.*Salvia miltiorrhiza* Bunge [Lamiaceae] (Danshen), 200g  5.*Achyranthes bidentata* Blume [Amaranthaceae](Niuxi), 125g  6.*Ophiopogon japonicus* (Thunb.) Ker Gawl.[Asparagaceae](Maidong), 125g  7.*Polygonatum sibiricum* Redouté [Asparagaceae](Huangjing), 125g  8.*Pueraria montana var.lobata* (Willd.) Maesen & S.M.Almeida ex Sanjappa & Predeep [Fabaceae](Gegen), 125g  9.*Morus alba* L. [Moraceae](Sangye), 125g  10.*Coptis chinensis* Franch. [Ranunculaceae](Huanglian), 41.7g   1. *Epimedium sagittatum* (Siebold & Zucc.) Maxim. [Berberidaceae]   (Yinyanghuo), 166.7g  Water extracted to clear paste, add about 167g of micronized silica gel, dry and put into capsules and make 1000 capsules, 0.5g per capsule | Y-Prepared  according to Chinese Pharmacopoeia (2020 Edition) | Y –  HPLC-ELSD (64),  HPLC-DAD (65) |
