# Supplementary material for: Treatment options of traditional Chinese patent medicines for dyslipidemia in patients with prediabetes: A systematic review and network meta-analysis
Source: Front Pharmacol. 2022 Aug 29;13:942563. doi: 10.3389/fphar.2022.942563 (PMC9465834; doi:10.3389/fphar.2022.942563)
Supplement: Supplementary file 9 [file Table5.DOCX]

| **Table 5** Final classification of 8 interventions for prediabetes | | | | |  |  |
| --- | --- | --- | --- | --- | --- | --- |
| ΔLDL-C | | | | |  |  |
| Certainty of the evidence | Category | Intervention | Intervention vs.LM  SMD (95% CI) | SUCRA |  |  |
| High certainty (moderate to high certainty evidence) | Category 1: among the most effective | none |  |  |  |  |
|  | Category 0: among the least effective | none |  |  |  |  |
| Low certainty (low to very low certainty evidence) | Category 1: might be among the most effective | Jinlida+LM | -0.31 (-0.59,-0.04) | 0.79 |  |  |
|  | Category 0: might be among the least effective | Shenqi+LM | -0.34 (-0.73,0.05) | 0.79 |  |  |
|  |  | Tianmai+LM | -0.23 (-0.72,0.26) | 0.68 |  |  |
|  |  | Jinqi+LM | -0.17 (-0.64,0.31) | 0.62 |  |  |
|  |  | Tangmaikang+LM | -0.10 (-0.78,0.58) | 0.56 |  |  |
|  |  | Oral drugs+LM | -0.08 (-0.67,0.50) | 0.53 |  |  |
|  |  | Placebo+LM | 0.94 (0.21,1.67) | 0.09 |  |  |
|  |  | Tianqi+LM | 0.99 (0.19,1.79) | 0.05 |  |  |
| ΔTG |  |  |  |  |  |  |
| Certainty of the evidence | Category | Intervention | Intervention vs.LM  SMD (95% CI) | SUCRA |  |  |
| High certainty (moderate to high certainty evidence) | Category 1: among the most effective | Shenqi+LM | -0.49 (-0.85, -0.12) | 0.87 |  |  |
|  | Category 0: among the least effective | Jinlida+LM | -0.19 (-0.55, 0.17) | 0.61 |  |  |
| Low certainty (low to very low certainty evidence) | Category 1: might be among the most effective | Jinqi+LM | -0.44 (-0.81, -0.06) | 0.83 |  |  |
|  | Category 0: might be among the least effective | Tangmaikang+LM | -0.22 (-0.68, 0.24) | 0.65 |  |  |
|  |  | Tianmai+LM | -0.11 (-0.88, 0.66) | 0.56 |  |  |
|  |  | Oral drugs+LM | 0.03 (-0.52, 0.59) | 0.41 |  |  |
|  |  | Placebo+LM | 0.99 (-0.12, 2.10) | 0.11 |  |  |
|  |  | Tianqi+LM | 1.19 (-0.05, 2.42) | 0.05 |  |  |
| ΔTC | | | | |  |  |
| High certainty (moderate to high certainty evidence) | Category 1: among the most effective | Shenqi+LM | -0.51 (-0.86, -0.17) | 0.85 |  |  |
|  | Category 0: among the least effective | Jinlida+LM | -0.11 (-0.48, 0.27) | 0.49 |  |  |
| Low certainty (low to very low certainty evidence) | Category 1: might be among the most effective | Jinqi+LM | -0.44 (-0.80,-0.08) | 0.78 |  |  |
|  |  | none |  |  |  |  |
|  | Category 0: might be among the least effective | Tianmai+LM | -0.54 (-1.36,0.28) | 0.80 |  |  |
|  |  | Tangmaikang+LM | -0.31 (-0.77, 0.14) | 0.69 |  |  |
|  |  | Oral drugs+LM | 0.07 (-0.46,0.61) | 0.35 |  |  |
|  |  | placebo+LM | 0.96 (-0.19,2.11) | 0.13 |  |  |
|  |  | Tianqi+LM | 1.17 (-0.09,2.43) | 0.05 |  |  |
| ΔHDL-C | | | | |  |  |
| High certainty (moderate to high certainty evidence) | Category 1: among the most effective | none |  |  |  |  |
|  | Category 0: among the least effective | Tianmai+LM | 0.05 (-0.23,0.33) | 0.53 |  |  |
| Low certainty (low to very low certainty evidence) | Category 1: might be among the most effective | Shenqi+LM | 0.29 (0.06,0.51) | 0.89 |  |  |
|  |  | Jinqi+LM | 0.16 (0.01,0.31) | 0.73 |  |  |
|  | Category 0: might be among the least effective | Tangmaikang+LM | 0.26 (-0.19,0.70) | 0.81 |  |  |
|  |  | Jinlida+LM | 0.10 (-0.04,0.24) | 0.61 |  |  |
|  |  | Oral drugs+LM | -0.01 (-0.32,0.30) | 0.41 |  |  |
|  |  | Tianqi+LM | -0.47 (-0.93, -0.01) | 0.08 |  |  |
|  |  | Placebo+LM | -0.49 (-0.91, -0.07) | 0.06 |  |  |
