# Supplementary material for: Treatment options of traditional Chinese patent medicines for dyslipidemia in patients with prediabetes: A systematic review and network meta-analysis
Source: Front Pharmacol. 2022 Aug 29;13:942563. doi: 10.3389/fphar.2022.942563 (PMC9465834; doi:10.3389/fphar.2022.942563)
Supplement: Supplementary file 10 [file DataSheet5.PDF]

Supplemental file 5

Ranking probability of 6 CTPMs on ΔTG、ΔTC、ΔHDL-C and ΔLDL-C

Treatment	ΔTG		ΔTC		ΔHDL-C		ΔLDL-C	
	SUCRA	Rank	SUCRA	Rank	SUCRA	Rank	SUCRA	Rank
Shenqi	87.3	1	84.5	1	89.1	1	79.2	1
Tianmai	55.7	5	79.8	2	52.5	5	67.5	3
Tianqi	4.5	9	4.6	9	8.4	8	5.4	9
Jinqi	83.5	2	78.3	3	72.6	3	62.1	4
Jinlida	61.4	4	49.2	5	61.4	4	78.5	2
Tangmaikang	65.1	3	68.9	4	80.5	2	55.5	5
LM	39.9	7	37.5	6	38.9	7	40.8	7
placebo	11.1	8	12.7	8	6	9	8.6	8
western oral drug	40.6	6	34.7	7	40.6	6	52.5	6

Note:Ranking: probability of being the best treatment, of being the second best. the third best and so on,among the 6 treatments.Shenqi: Shen qi jiang tang capsule/granule、Tianmai: Tian mai xiao ke tablet、Tianqi: Tian qi capsule、Jinqi: Jin qi jiang tang tablet、Jinlida: Jin li da granule、Tangmaikang: Tang mai kang granule; FBG: fasting blood glucose; PBG: postprandial blood glucose. LM: lifestyle modification; SUCRA: surface under the cumulative ranking curve
