# Supplementary material for: Treatment options of traditional Chinese patent medicines for dyslipidemia in patients with prediabetes: A systematic review and network meta-analysis
Source: Front Pharmacol. 2022 Aug 29;13:942563. doi: 10.3389/fphar.2022.942563 (PMC9465834; doi:10.3389/fphar.2022.942563)
Supplement: Supplementary file 11 [file Table3.DOCX]

**Table 3** Potential mechanisms of 6 TCPM on prediabetes and T2DM in vivo experiments

| Formulation | References | Beneficial effects | Potential mechanisms |
| --- | --- | --- | --- |
| Shenqi capsule/granule | Shi S et al(2021) | Improving insulin sensitivity | Decreasing the mRNA expression level and serum concentration of inflammatory cytokines such as TNF-αIL-6, and IL-1β, suppressing the p-NFκB protein over-expression, up-regulating protein expression of p-Akt and GLUT2 in a rat model of insulin resistance(66) |
|  | Zhang Q et al(2019) | Kidney protection | Reducing caspase-3-positive cells in diabetic kidneys, upregulating Bcl-2 and regucalcin expressions and reducing casp3 and Apaf1 expressions in diabetic rats (67) |
| Tianmai tablet | Wang N et al(2016) | Improving insulin sensitivity | Decreasing IRS-1, IRS-2, PI3-K p85α, and AKT2 gene expression and also IRS-1, IRS-2, PI3-K, AKT2, and p-AKT2 protein expression levels through the PI3K/AKT pathway in diabetic rats (68) |
|  | Zhang Q et al(2014) | Reducing fasting glucose level | Decreasing levels of forkhead box O3 (FoxO3), phosphoenolpyruvate carboxykinase 2 (Pck2), and protein tyrosine phosphatase 1B (Ptp1b), increasing v-akt murine thymoma viral oncogene homolog 1 (Akt1) and insulin receptor substrate 2 (Irs2) through insulin signaling pathway in diabetic rats (69) |
|  | Zhang Q et al(2014) | Activating insulin synthesis | Increasing the expression of miR-375 and miR-30d in diabetic rats (70) |
| Tianqi capsule | Zhang SX et al(2010) | Improving glucose  metabolism | Down-regulating the apolipoprotein E, apolipoprotein A-I, Ig gamma-2A chain C region, up-regulating transthyretin (TTR), haptoglobin (Hp), serum amyloid p-componen (SAP) and prothrombin in diabetic rats (71) |
|  | Li X et al(2013) | Preventing diabetes | Reducing the "G" allele frequencies of rs1142345 (A>G) in the thiopurine S-methyltransferase (TPMT) gene in prediabetic patients (72) |
| Jinqi tablet | Qian Q et al(2012)  Liu Q et al(2017)  Lv YJ et al(2017) | Improving insulin sensitivity | Up-regulating the expression of IRS-1 in the liver and IRS-2 in the skeletal muscles of the KK-Ay mice (73); inhibiting the phosphorylation of JNK,ERK1/2 and p38 (74); lowering the circulating T helper 17 (Th17) frequencies, serum interleukin-17 (IL-17) and interleukin-23 (IL-23) levels in diabetic SD rats (75) |
|  | Liu Q et al(2017)  Zhao HL et al(2012)  Qian Q et al(2012)  Gao LH et al(2013) | Increasing glucose uptake and glycogen synthesis | elevating the insulin-stimulated glucose uptake with upregulated phosphorylation of AKT in PA induced insulin resistant L6 myotubes (74); downregulating miRNA-29b and targeting AKT (76); increasing the expression of AMPK in the liver and muscular tissues and the GLUT-4 in the skeletal muscles, reversing the decreased glycogen level in liver (73,77) |
|  | Qian Q et al(2012)  Zhao HL et al(2012)  Liu Q et al(2017) | Enhancing lipid metabolism | Increasing the expression and tyrosine phosphorylation of AMPK (73); decreasing the expression of acetyl CoA carboxylase (ACC), fatty acid synthase (FAS) and hormone sensitive lipase (HSL) in diabetic KK-Ay mice (74,76) |
| Jinlida granule | Zhou HR et al(2022)  Zhang H et al(2019) | Enhancing lipid metabolism | Increasing the expression of the thermogenic protein, UCP1, in the beige adipose tissue of mice, inhibiting the expression of miR-27a in X9 cells thereby promoting thermogenesis in beige adipocytes (78); activating the brown adipose tissue thermogenesis via enhancement of mitochondrial biogenesis and fatty acid oxidation metabolism (79) |
|  | Wang C et al(2018) | Improving dysfunction of Hypothalamic-Pituitary-  Thyroid Axis | Increasing the levels of serum T3 and T4, TR mRNA in liver tissue, TSHR, and NIS mRNA in thyroid tissue, decreasing the levels of Dio1 mRNA, pI-κB, pNF-κB, TNFα and IL-6 in diabetic rats (80) |
|  | Jin X et al(2015)  Zang SS et al(2015)  Liu Y et al(2015) | Improving insulin sensitivity | Increasing the expression of insulin receptor substrate (IRS-1) mRNA and protein, alleviating the expression of diacylglycerol acyltransferase (DGAT) in skeletal muscle (81); increasing AMPK and acetyl-CoA carboxylase (ACC) phosphorylation in skeletal muscle; reducing hepatic oxidative stress through reducing phosphorylation protein levels of JNK and p38MAPK (82-83) |
| Tangmaikang granule | Chen Q et al(2017) | Reducing insulin resistance | Reducing hepatic lipid accumulation and lowering levels of serum inflammatory factor CRP (84) |
