# Supplementary material for: Treatment options of traditional Chinese patent medicines for dyslipidemia in patients with prediabetes: A systematic review and network meta-analysis
Source: Front Pharmacol. 2022 Aug 29;13:942563. doi: 10.3389/fphar.2022.942563 (PMC9465834; doi:10.3389/fphar.2022.942563)
Supplement: Supplementary file 12 [file Table4.DOCX]

**Table 4a** the league table of ΔLDL-C and ΔHDL-C

| Comparisons for *Δ*LDL-C (bottom left) and *Δ*HDL-C (upper right) of the 6 TPCM. | | | | | | | | |
| --- | --- | --- | --- | --- | --- | --- | --- | --- |
| basic | **0.61 (0.40,0.93)** | 0.99 (0.72,1.35) | **1.33 (1.07,1.67)** | 1.05 (0.80,1.39) | **0.63 (0.39,0.99)** | **1.17 (1.01,1.36)** | 1.10 (0.96,1.27) | 1.30 (0.83,2.02) |
| 0.39 (0.19,0.81) | placebo | 1.61 (0.96,2.72) | **2.18 (1.35,3.51)** | **1.72 (1.25,2.35)** | 1.02 (0.84,1.24) | **1.91 (1.22,2.99)** | **1.80 (1.16,2.81)** | **2.12 (1.15,3.90)** |
| 1.09 (0.60,1.95) | **2.78 (1.09,7.11)** | oral  drugs | 1.35 (0.92,1.98) | 1.06 (0.70,1.61) | 0.63 (0.36,1.11) | 1.18 (0.84,1.67) | 1.12 (0.85,1.47) | 1.31 (0.95,1.80) |
| 1.40 (0.95,2.07) | **3.60 (1.57,8.26)** | 1.29 (0.64,2.61) | Shenqi | 0.79 (0.55,1.13) | **0.47 (0.28,0.79)** | 0.88 (0.67,1.15) | 0.83 (0.64,1.08) | 0.97 (0.59,1.60) |
| 1.26 (0.77,2.05) | **3.22 (1.86,5.58)** | 1.16 (0.54,2.49) | 0.90 (0.48,1.67) | Tianmai | 0.59 (0.41,0.86) | 1.11 (0.81,1.53) | 1.05 (0.77,1.44) | 1.23 (0.73,2.08) |
| 0.37 (0.17,0.82) | 0.95 (0.69,1.30) | **0.34 (0.13,0.92)** | **0.26 (0.11,0.64)** | **0.29 (0.16,0.55)** | Tianqi | **1.87 (1.15,3.05)** | **1.77 (1.09,2.87)** | **2.07 (1.09,3.94)** |
| 1.18 (0.73,1.90) | **3.02 (1.26,7.25)** | 1.09 (0.51,2.32) | 0.84 (0.45,1.55) | 0.94 (0.47,1.86) | **3.18 (1.26,8.06)** | Jinqi | 0.94 (0.77,1.16) | 1.11 (0.69,1.77) |
| **1.37 (1.04,1.80)** | **3.50 (1.60,7.67)** | 1.26 (0.75,2.11) | 0.97 (0.60,1.57) | 1.09 (0.62,1.90) | **3.68 (1.58,8.59)** | 1.16 (0.67,2.01) | Jinlida | 1.17 (0.77,1.79) |
| 1.10 (0.56,2.17) | **2.83 (1.04,7.67)** | 1.02 (0.73,1.42) | 0.79 (0.36,1.72) | 0.88 (0.38,2.02) | **2.98 (1.04,8.49)** | 0.94 (0.41,2.15) | 0.81 (0.44,1.50) | Tangmaikang |
| Note: Data of comparisons for the *Δ*LDL-C and *Δ*HDL-C are SMD (95% CI). The 95% confidence interval which don’t range across 1 favors the column-defining treatment and are showed in bold. | | | | | | | | |

**Table 4b** the league table of ΔTG and ΔTC

| Comparisons for ΔTG(bottom left ) and ΔTC(upper right) of the 6 TPCM. | | | | | | | | |
| --- | --- | --- | --- | --- | --- | --- | --- | --- |
| basic | 2.61 (0.83,8.23) | 1.08 (0.63,1.84) | **0.60 (0.42,0.84)** | 0.58 (0.26,1.33) | 3.22 (0.91,11.41) | **0.65 (0.45,0.92)** | 0.90 (0.62,1.31) | 0.73 (0.46,1.15) |
| 0.37 (0.12,1.13) | placebo | 0.41 (0.12,1.46) | **0.23 (0.07,0.76)** | **0.22 (0.10,0.50)** | 1.23 (0.73,2.09) | **0.25 (0.07,0.82)** | 0.34 (0.10,1.15) | **0.28 (0.08,0.96)** |
| 0.97 (0.56,1.68) | 2.60 (0.75,8.99) | oral  drugs | 0.56 (0.29,1.05) | 0.54 (0.20,1.44) | 3.00 (0.76,11.82) | 0.60 (0.32,1.14) | 0.84 (0.48,1.45) | 0.68 (0.45,1.02) |
| **1.63 (1.13,2.35)** | 4.37 (1.36,14.10) | 1.68 (0.87,3.27) | Shenqi | 0.97 (0.40,2.38) | **5.39 (1.46,19.98)** | 1.08 (0.66,1.78) | 1.51 (0.90,2.51) | 1.22 (0.69,2.16) |
| 1.12 (0.52,2.42) | 3.00 (1.35,6.67) | 1.16 (0.45,2.99) | 0.69 (0.29,1.62) | Tianmai | **5.53 (2.12,14.44)** | 1.11 (0.45,2.72) | 1.54 (0.63,3.82) | 1.25 (0.49,3.21) |
| 0.30 (0.09,1.05) | 0.82 (0.48,1.41) | 0.32 (0.08,1.22) | 0.19 (0.05,0.68) | 0.27 (0.10,0.72) | Tianqi | 0.20 (0.05,0.74) | 0.28 (0.07,1.04) | 0.23 (0.06,0.87) |
| **1.55 (1.06,2.25)** | 4.16 (1.29,13.43) | 1.60 (0.82,3.12) | 0.95 (0.56,1.61) | 1.38 (0.59,3.27) | 5.07 (1.40,18.43) | Jinqi | 1.40 (0.83,2.33) | 1.13 (0.63,2.02) |
| 1.21 (0.84,1.73) | 3.25 (1.01,10.45) | 1.25 (0.71,2.20) | 0.74 (0.44,1.24) | 1.08 (0.46,2.54) | 3.96 (1.10,14.34) | 0.78 (0.47,1.31) | Jinlida | 0.81 (0.48,1.38) |
| 1.25 (0.79,1.98) | **3.35 (1.01,11.17)** | 1.29 (0.83,2.02) | 0.77 (0.43,1.38) | 1.12 (0.45,2.75) | **4.09 (1.10,15.29)** | 0.81 (0.45,1.46) | 1.03 (0.61,1.75) | Tangmaikang |
| Note: Data of comparisons for the ΔTG and ΔTC are SMD (95% CI). The 95% confidence interval which don’t range across 1 favors the column-defining treatment and are showed in bold. | | | | | | | | |
