# Supplementary figures and images for: Treatment options of traditional Chinese patent medicines for dyslipidemia in patients with prediabetes: A systematic review and network meta-analysis
Source: Front Pharmacol. 2022 Aug 29;13:942563. doi: 10.3389/fphar.2022.942563 (PMC9465834; doi:10.3389/fphar.2022.942563)

# Supplemental file 8 correction funnel plot

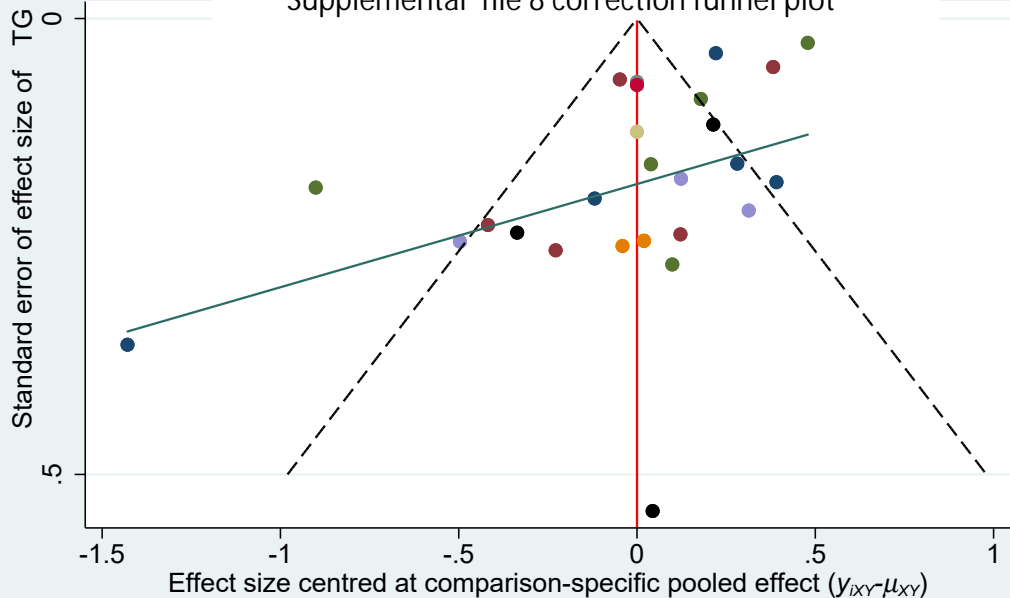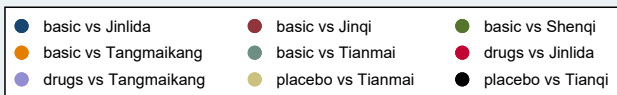

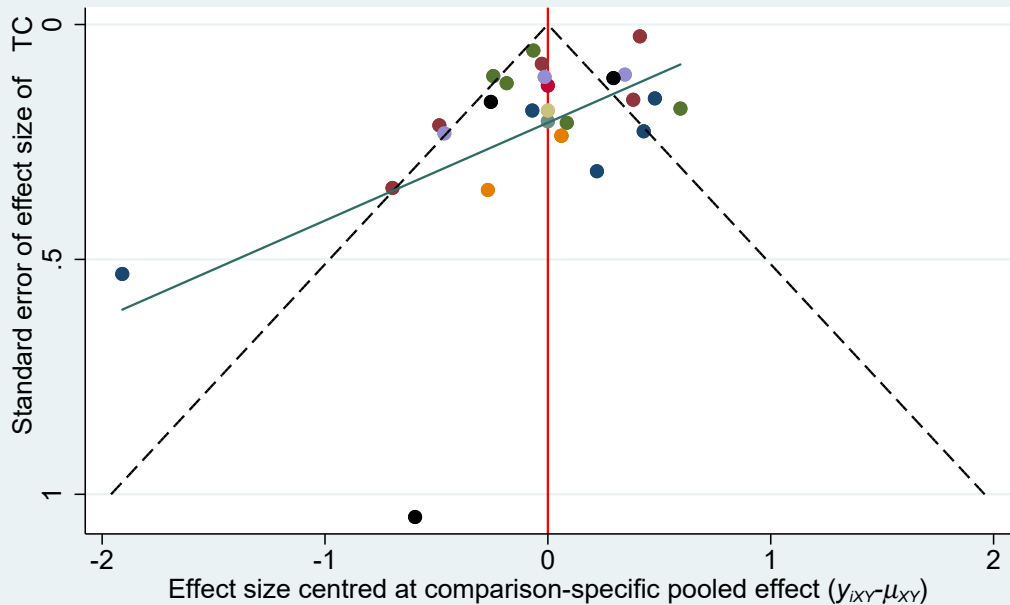

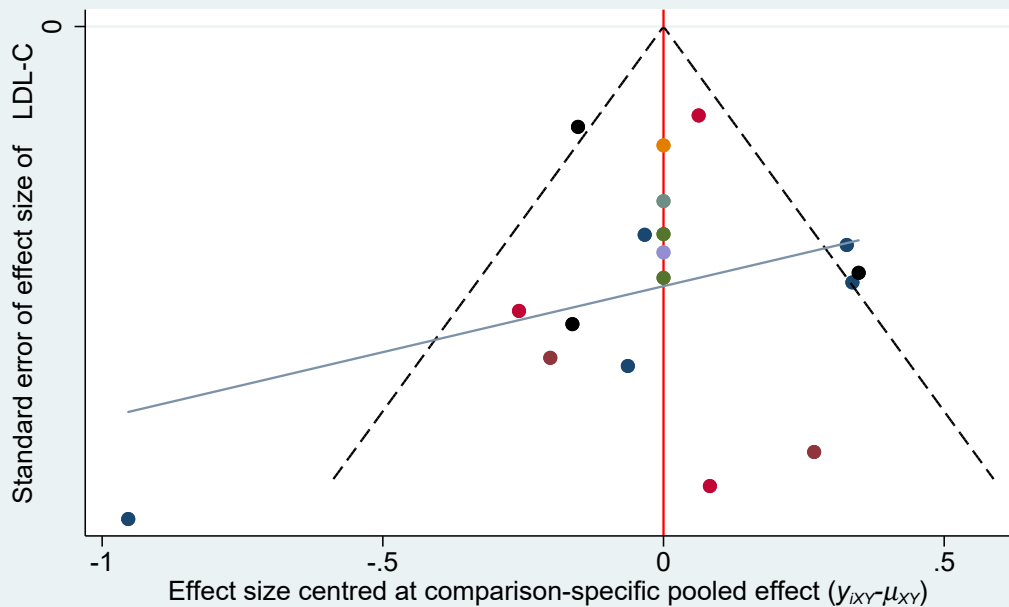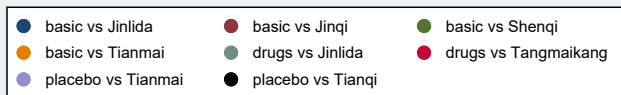

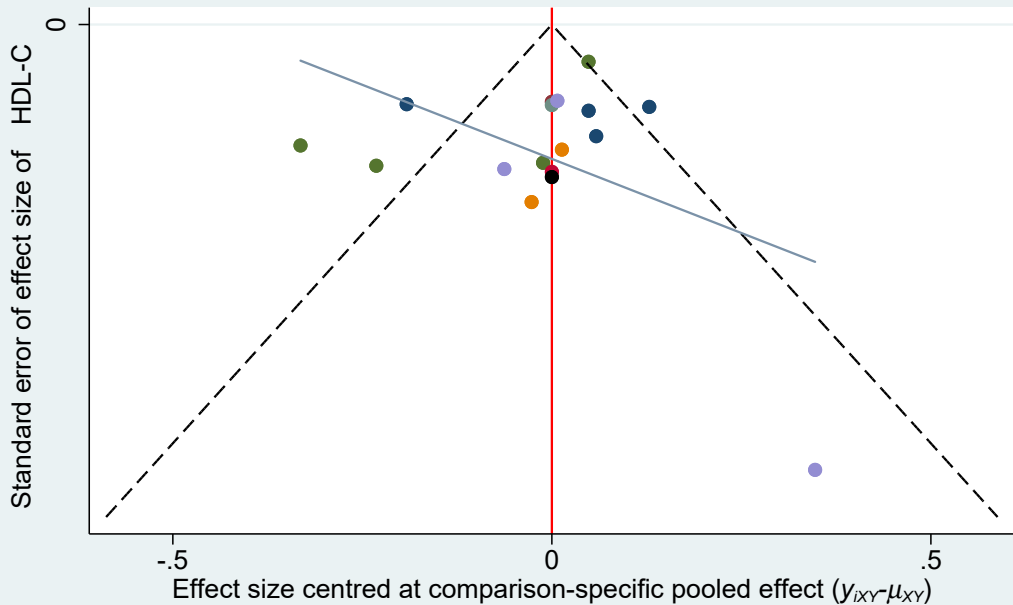

Supplement: Supplementary file 13 [file DataSheet8.PDF]
