# Supplementary material for: Treatment options of traditional Chinese patent medicines for dyslipidemia in patients with prediabetes: A systematic review and network meta-analysis
Source: Front Pharmacol. 2022 Aug 29;13:942563. doi: 10.3389/fphar.2022.942563 (PMC9465834; doi:10.3389/fphar.2022.942563)
Supplement: Supplementary file 14 [file Table6.DOCX]

Table 5 Meta-regression of LDL-C

| _ES | Coef. | Std. Err. | t | P>t | lower CI | upper CI |
| --- | --- | --- | --- | --- | --- | --- |
| treatment duration | 0.0657139 | 0.1628049 | 0.4 | 0.694 | -0.2926173 | 0.4240452 |
| type of TCPM | 0.0951485 | 0.0910892 | 1.04 | 0.319 | -0.1053375 | 0.2956346 |
| control group | 0.0392593 | 0.1306007 | 0.3 | 0.769 | -0.2481908 | 0.3267094 |
| diagnostic criteria | -0.2053687 | 0.1292378 | -1.59 | 0.14 | -0.4898192 | 0.0790818 |
| risk of bias | -0.1367099 | 0.1656466 | -0.83 | 0.427 | -0.5012955 | 0.2278757 |
| baseline of LDL-C | -0.4039831 | 0.2059021 | -1.96 | 0.076 | -0.8571704 | 0.0492043 |
| _cons | 0.6724347 | 0.729448 | 0.92 | 0.376 | -0.9330695 | 2.277939 |
| Meta-regression: Number of obs = 18  REML estimate of between-study variance: tau^2^ = 0.1131  % residual variation due to heterogeneity: I-squared_res = 71.31%  Proportion of between-study variance explained: Adj R-squared = 29.52%  Joint test for all covariates: Model F(6,11) = 1.8  With Knapp-Hartung modification: Prob > F = 0.1878 | | | | | | |
